# Combining metabolite doping and metabolic engineering to improve 2-phenylethanol production by engineered cyanobacteria

**DOI:** 10.3389/fbioe.2022.1005960

**Published:** 2022-09-20

**Authors:** Giulia Usai, Alessandro Cordara, Angela Re, Maria Francesca Polli, Giuseppe Mannino, Cinzia Margherita Bertea, Debora Fino, Candido Fabrizio Pirri, Barbara Menin

**Affiliations:** ^1^ Centre for Sustainable Future Technologies, Fondazione Istituto Italiano di Tecnologia, Turin, Italy; ^2^ Department of Applied Science and Technology—DISAT, Politecnico di Torino, Turin, Italy; ^3^ Department of Agricultural, Forest and Food Sciences—DISAFA, University of Turin, Grugliasco, Italy; ^4^ Plant Physiology Unit, Department of Life Sciences and Systems Biology, University of Turin, Turin, Italy

**Keywords:** cyanobacteria, photosynthesis, aromatics, 2-phenylethanol, phenylalanine, metabolite doping, shikimate pathway, shikimate kinase

## Abstract

2-Phenylethanol (2-PE) is a rose-scented aromatic compound, with broad application in cosmetic, pharmaceutical, food and beverage industries. Many plants naturally synthesize 2-PE via Shikimate Pathway, but its extraction is expensive and low-yielding. Consequently, most 2-PE derives from chemical synthesis, which employs petroleum as feedstock and generates unwanted by products and health issues. The need for “green” processes and the increasing public demand for natural products are pushing biotechnological production systems as promising alternatives. So far, several microorganisms have been investigated and engineered for 2-PE biosynthesis, but a few studies have focused on autotrophic microorganisms. Among them, the prokaryotic cyanobacteria can represent ideal microbial factories thanks to their ability to photosynthetically convert CO_2_ into valuable compounds, their minimal nutritional requirements, high photosynthetic rate and the availability of genetic and bioinformatics tools. An engineered strain of *Synechococcus elongatus* PCC 7942 for 2-PE production, i.e., p120, was previously published elsewhere. The strain p120 expresses four heterologous genes for the complete 2-PE synthesis pathway. Here, we developed a combined approach of metabolite doping and metabolic engineering to improve the 2-PE production kinetics of the *Synechococcus elongatus* PCC 7942 p120 strain. Firstly, the growth and 2-PE productivity performances of the p120 recombinant strain were analyzed to highlight potential metabolic constraints. By implementing a BG11 medium doped with L-phenylalanine, we covered the metabolic burden to which the p120 strain is strongly subjected, when the 2-PE pathway expression is induced. Additionally, we further boosted the carbon flow into the Shikimate Pathway by overexpressing the native Shikimate Kinase in the *Synechococcus elongatus* PCC 7942 p120 strain (i.e., 2PE_*aroK*). The combination of these different approaches led to a 2-PE yield of 300 mg/gDW and a maximum 2-PE titer of 285 mg/L, 2.4-fold higher than that reported in literature for the p120 recombinant strain and, to our knowledge, the highest recorded for photosynthetic microorganisms, in photoautotrophic growth condition. Finally, this work provides the basis for further optimization of the process aimed at increasing 2-PE productivity and concentration, and could offer new insights about the use of cyanobacteria as appealing microbial cell factories for the synthesis of aromatic compounds.

## 1 Introduction

The global emergency to reduce greenhouse gas emissions to net-zero is currently a burning issue. As part of the Paris Agreement and the European Green Deal, the transition to a climate-neutral society should be gained by investigating realistic technological solutions, by aligning action in key areas such as industrial policy, finance and research (Delbeke and Vis, 2019; European Commission, 2019). In this panorama, biorefineries can help decarbonization by fostering greener solutions. Among these, the photosynthetic activity of some organisms can be outstandingly fitting. Indeed, plants, algae and cyanobacteria can naturally convert CO_2_, when sunlight and water are both present, to organic molecules, useful as fuels or other add-value commodities (Daneshvar et al., 2022). The prokaryotic cyanobacteria, which fix CO_2_ more efficiently than higher plants and grow faster (Li et al., 2008), are one of the most promising actors that can deal with this time in history. Indeed, cyanobacteria can grow in non-arable lands and in nutritionally poor conditions (Jenkins, 1991; Ducat, Way and Silver, 2011) or non-potable water (Nozzi, Oliver and Atsumi, 2013; Sánchez-Bayo et al., 2020). Additionally, in comparison with eukaryotic algae and plants, cyanobacteria are more genetically amenable (Berla et al., 2013; Lau, Matsui and Abdullah, 2015; Sengupta et al., 2019; Wang, Gao and Yang, 2020) and so attractive hosts for challenging metabolic engineering strategies (Bishé, Taton and Golden, 2019; Santos-Merino, Singh and Ducat, 2019; Vasudevan et al., 2019). Indeed, they have been largely metabolically engineered to produce a huge array of valuable products (Zhou and Li, 2010; Lai and Lan, 2015; Santos-Merino, Singh and Ducat, 2019).

Recently, some efforts have been targeted to produce aromatic compounds by cyanobacteria (Ni et al., 2018; Xue and He, 2018; Huccetogullari, Luo and Lee, 2019; Brey et al., 2020; Jin et al., 2021). Aromatics, characterized by a benzene ring or derivatives, are mainly adopted as organic solvents, dyes and precursors for the synthesis of many products employed in the food, pharmaceutical and chemical fields. Currently, the majority of aromatic chemicals are manufactured using petroleum as the raw source, which makes them undesirable for environmental, safety and health issues (Gosset, 2009; Qian et al., 2019).

In all microorganisms and plants the aromatics synthesis takes place via the Shikimate Pathway (SKP), a sequence of seven enzymatic steps in which phosphoenolpyruvate and erythrose 4-phosphate are converted to chorismate, the precursor of the three aromatic amino acids (AAA) - tryptophan (Trp), phenylalanine (Phe), and tyrosine (Tyr) - and most of the aromatic metabolites. Several intermediates can serve for the subsequent synthesis of both aromatic and non-aromatic molecules commercially valuable (Herrmann, 1995; Mir, Jallu and Singh, 2015).

*De novo* synthesis of aromatic compounds has been already achieved through different organisms by exploiting the SKP (Dickey, Forti and Kunjapur, 2021). However, the enzymes involved in some steps of the SKP are tightly regulated by feedback inhibition and transcriptional mechanisms, which hamper their use for target compounds oversynthesis. In bacteria and yeast, one of the most studied regulatory mechanisms is the 3-Deoxy-D-arabinoheptulosonate 7-phosphate (DAHP) synthase (EC 2.5.1.54), the first committed enzyme of the SKP. *E. coli* expresses three genes encoding DAHP synthases, which are respectively sensitive to a specific AAA (Jossek, Bongaerts and Sprenger, 2001; Chávez-Béjar et al., 2012; Noda and Kondo, 2017). Furthermore, as already reported in some bacteria and yeast, the shikimate kinase (EC 2.7.1.71), which catalyzes the conversion of shikimate in shikimate-3-phosphate (the fifth step in SKP), can relevantly affect the carbon flow by acting as bottleneck in the SKP (Luttik et al., 2008; Takai et al., 2009; Rodriguez et al., 2015; Xu et al., 2019).

So far, numerous efforts have been already pursued to yield aromatic chemicals by genetically and non-genetically modified microorganisms. 2-phenylethanol (2-PE) is one of the most valuable aromatic compounds, with a global market value estimated to reach 351 Million USD by 2027 (Kang et al., 2014; Kim, Lee and Oh, 2014; Romagnoli et al., 2015; Averesch and Krömer, 2018). 2-PE is an aromatic alcohol with pleasant rose-like scent, which makes it exploitable mostly in the food, fragrance, and flavor industries, but still employable as antifungal or fuel additive (Etschmann et al., 2002; Majdabadi et al., 2018; Nielson, Machas and Mckenna, 2018; Fang and Sung, 2021). Plant-extracted 2-PE costs 1,000 US$/kg and suffers from economics and scalability for a sustainable industrial production (Liu et al., 2018; Qian et al., 2019). On the other hand, biotechnologically synthesized 2-PE has a value of around 220 US$/kg (Mitri et al., 2022), so the 2-PE synthesis by microorganisms has been emerging as appealing bioproduction system, alternatively to plant extraction (Gu et al., 2020; Kong et al., 2020; Dai et al., 2021; Zhu et al., 2021).

While a few bacteria are known to naturally synthesize 2-PE (Zhang et al., 2014a; Liu et al., 2018a; Liu et al., 2018b), the microbiological 2-PE biosynthesis has been intensively investigated mainly in yeast. Particularly, yeast preferentially use the three-stepped Ehrlich pathway for 2-PE production, by starting from phenylalanine. Thus, to sustain 2-PE production, the yeast-based microbial platform needs to be fed with both sugar substrate and aromatic amino acid (Liu et al., 2019a; Gu et al., 2020; Dai et al., 2021). Generally, this strategy is known as co-substrate feeding or metabolite doping (Liu et al., 2019b; Hong et al., 2019; Park et al., 2019; Gu et al., 2020; Liu, Santala and Stephanopoulos, 2020; Chang et al., 2021). However, single substrate feeding is conventionally preferred but naturally elicits stoichiometric constraints on available carbon atoms, energy and redox state, leading to metabolic imbalance, when a specific route has to be exploited. Also, in cyanobacterial and microalgal cultivations, co-substrate feeding or nutrient modulation strategies have been adopted (Papazi et al., 2012; Anfelt et al., 2015; Ndayisenga et al., 2021). In comparison to carbohydrate-using microorganisms, cyanobacteria may represent a more sustainable alternative because, drawing energy from photosynthesis, they avoid expensive fermentable sugars as carbon feedstock and can be exploited for CO_2_ capture and conversion. Furthermore, cyanobacteria have been already proved to be suitable hosts for 2-PE production (Ni et al., 2018). In a previous work, Ni and co-workers applied a synthetic biology approach aimed at redirecting the carbon flux toward the SKP for 2-PE synthesis in *Synechococcus elongatus* PCC 7942 (*S. elongatus* PCC 7942) (Ni et al., 2018). They have successfully obtained a mutant strain (p120), which heterologously overexpresses four genes necessary for 2-PE biosynthesis: *aroG* ^*fbr*^ and *pheA* ^*fbr*^, related to the SKP, and *kivD* and *adhA* to effectively synthetize 2-PE. Both the protein products of *aroG* and *pheA* are known bottlenecks afflicting the SKP, and therefore these gene sequences have been genetically modified not to be sensitive to the feedback inhibition by phenylalanine (feedback inhibition resistant, *fbr*). Phenylpyruvate decarboxylase is the protein product of *kivD* and catalyzes the conversion of phenylpyruvate in phenylacetaldehyde. Finally, the alcohol dehydrogenase A, the protein encoded by *adhA* gene, converts phenylacetaldehyde in 2-PE. Particularly, in Ni et al., 2018 the p120 mutant strain produced 119.5 mg/L 2-PE in 10 days (Ni et al., 2018). However, we were not able to replicate this outcome in our laboratory.

In the present work, we optimized the 2-PE production process in the recombinant *S. elongatus* PCC 7942 p120 strain (Ni et al., 2018) by combining two strategies of metabolite doping and metabolic engineering: 1) we implemented a phenylalanine-doped growth medium to overcome metabolic load and the competition between 2-PE synthetic pathway and endogenous routes for Phe usage; 2) we metabolically engineered the p120 mutant strain to increase the carbon flow into the SKP by means of the endogenous Shikimate Kinase overexpression, obtaining a new engineered strain (2PE_*aroK*). The resulting mutant strain and the related biosystem could effectively increase 2-PE production and offer new insights about the aromatic metabolism in cyanobacteria.

## 2 Material and methods

### 2.1 Cyanobacterial strains, media and cultural conditions

All strains used in the present study are listed in Table 1. *Synechococcus elongatus* PCC 7942 was obtained from the Pasteur Culture Collection of Cyanobacteria and was cultivated in sterilized BG11 medium (Stanier et al., 1971) with an optimized recipe and some modifications (Cordara et al., 2018; Vasile et al., 2021). Briefly, the final BG11 medium composition is: 1.5 g/L NaNO_3_, 0.075 g/L MgSO_4_·7H_2_O, 0.004 g/L FeCl_3_·6H_2_O, 0.04 g/L K_2_HPO_4_, 0.036 g/L CaCl_2_, 0.024 g/L Na_2_EDTA·2H_2_O, 2.86 mg/L H_3_BO_3_, 1.81 mg/L MnCl_2_·4H_2_O, 0.39 mg/L Na_2_MoO_4_·2H_2_O, 0.22 mg/L ZnSO_4_·7H_2_O, 0.05 mg/L CuSO_4_·5H_2_O, 0.03 mg/L Co(NO_3_)_2_·6H_2_O. The BG11 medium was buffered with 6.05 g/L TES (2-([Tris(hydroxymethyl)methyl]amino)ethane-1-sulfonic acid sodium salt) to pH = 8. For the *S. elongatus* recombinant strains (*S. elongatus* p120, *S. elongatus* 7942_*aroK* and *S. elongatus* 2PE_*aroK*), the BG11 medium was supplemented with the appropriate antibiotic (20 μg/ml spectinomycin and/or 10 μg/ml kanamycin). Cells were grown either in shaking flasks at 30°C under continuous lighting (30 µmol photons m^−2^ s^−1^) 130 rpm or on solid BG11 1.5% agar plates. For the study of the physiological effect of aromatic compounds on the p120 mutant strain metabolism, the three aromatic amino acid (L-Phe, L-Tyr and L-Trp) and two aromatic vitamins (folic acid and cyanocobalamin) were supplemented into the BG11 medium individually or in combination. The toxicity assay was conducted by exposing the wild type strain to 0.1, 0.3, 0.5 and 1 g/L 2-PE. The growth of the all strains of *S. elongatus* used in this work were daily monitored by measuring optical density at 730 nm (OD_730_) and subsequently converted into biomass as gram of dry cell weight per liter (gDW/L), by using a conversion factor of 0.26 (previously achieved in our laboratory). Specific growth rate (µ, day^−1^) was obtained from the slope of the logarithmic plot of OD_730_ versus time, for the growth curve points enclosed into the exponential phase. Gene expression in mutant strains was inducted by adding 1 mM IPTG (Isopropyl β-d-1-thiogalactopyranoside) into the BG11 medium when the cell cultures reached OD_730_ = 1. One-way ANOVA or unpaired two-tailed *t*-test were used to assess the statistical significance of the cultural conditions screened in the present study (*p*-value < 0.05).

**TABLE 1:** Genetic features of plasmid vectors and strains used in the present work. *lacI* ^*q*^, LacI repressor gene; *kivD*, gene encoding phenylpyruvate decarboxylase; *adhA*, gene for alcohol dehydrogenase A; *aroG* ^*fbr*^, feedback-inhibition resistant DAHP synthase; *pheA* ^*fbr*^, feedback-inhibition-resistant chorismate mutase/prephenate dehydratase; *Spec* ^*R*^, spectinomycin-resistance cassette; *aroK*, endogenous gene for Shikimate Kinase expression in *S. elongatus*; Kan^R^, kanamycin-resistance cassette; P_trc_, IPTG-inducible promoter; NSI, homologous regions for Neutral Site I insertion; NSII, homologous regions for Neutral Site II insertion.

Plasmid or strain	Genetic features	Source
p120	lacI^q^; P_trc_::*kivD*, P_trc_::*aroG* ^*fbr*^, P_trc_::*pheA* ^*fbr*^ *,* P_trc_::*adhA*, Spec^R^, NSI homologous sites	Ni et al. (2018)
pAM2991	lacI^q^, P_trc_, Spec^R^, NSI homologous sites	addgene, https://www.addgene.org/40248/
pAM1579	Kan^R^, NSII homologous sites	addgene, https://www.addgene.org/40240/
pAM1579_LacI	lacI^q^, P_trc_, Kan^R^, NSII homologous sites	This work
pAM1579_LacI_*aroK*	lacI^q^, P_trc_,::*aroK*, Kan^R^, NSII homologous sites	This work
*S. elongatus* PCC 7942	wild type	Pasteur Culture Collection
*S. elongatus* p120	*S. elongatus* overexpressing 2-PE biosynthetic pathway enzymes (p120)	Ni et al. (2018)
*S. elongatus 7942_aroK*	*S. elongatus* overexpressing the native shikimate kinase gene	This work
*S. elongatus* 2PE_*aroK*	*S. elongatus* overexpressing both 2-PE biosynthetic pathway enzymes (p120) and the native shikimate kinase gene	This work

### 2.2 Plasmid construction and transformation

**FIGURE 1:**
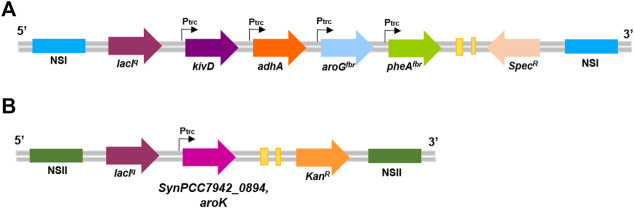
Composition of genetic vectors for heterologous 2-phenylethanol synthesis in *S. elongatus* PCC 7942 genome. **(A)** p120 vector contains: NSI (blue), homologous regions for chromosomal integration into the Neutral Site I; lacI^q^ (purple), LacI repressor gene; *kivD* (violet), gene encoding phenylpyruvate decarboxylase; *adhA* (orange), gene for alcohol dehydrogenase A; *aroG* ^*fbr*^ (light blue), feedback-inhibition resistant DAHP synthase; *pheA* ^*fbr*^ (green), feedback-inhibition-resistant chorismate mutase/prephenate dehydratase; *Spec* ^*R*^ (pink), spectinomycin-resistance cassette. **(B)** *aroK* overexpression vector: NSII (dark green), homologous regions for chromosomal integration into the Neutral Site II; *aroK* (dark pink), endogenous gene for shikimate kinase expression in *S. elongatus* (locus tag, SynPCC7942_0894); Kan^R^ (yellow), kanamycin-resistance cassette; P_trc_ (black arrow), IPTG-inducible promoter.

Chemically competent *Escherichia coli* DH5α strain was used for gene cloning and was grown in LB (Luria-Bertani) broth at 37°C, either in liquid cultures with continuous shaking (130 rpm) or on solid 1.5% (w/v) agar plates with the proper selection antibiotic (100 μg/ml spectinomycin or 50 μg/ml kanamycin). The plasmid p120 (kindly provided by Prof. Ping Xu at the State Key Laboratory of Microbial Metabolism of the Shanghai Jiao Tong University (Ni et al., 2018); Figure 1A and Table 1) is responsible for the heterologous expression of the pathway for 2-PE biosynthesis in *S. elongatus*. It contains two flanking sequences for targeted multigene insertion by homologous recombination in *S. elongatus* Neutral Site I (NSI). Also, the IPTG-inducible gene expression platform (*LacI* ^*q*^ repressor gene, LacI operator), spectinomycin-resistance cassette (aminoglycoside adenylyltransferase) and the four genes involved in 2-PE synthesis: feedback-inhibition resistant DAHP synthase (*aroG* ^*fbr*^, from *E. coli*), feedback-inhibition resistant chorismate mutase/prephenate dehydratase (*pheA* ^*fbr*^, from *E. coli*), phenylpyruvate decarboxylase (*kivD*, from *L. lactis* codon optimized for *S. elongatus*), and alcohol dehydrogenase A (*adhA*, from *Synechocystis* sp. PCC 6803) (Figure 1A). Each gene is preceded by trc promoter, lacI operator and RBS. Additionally, the pAM1579-LacI was created by standard cloning LacI repressor gene, LacI operator, trc promoter and RBS from pAM2991 into pAM1579 (Ptrc start-NheI-FW CAT​GGCT​AGCGTA​CCG​AGC​TCG​AAT​TCC​ATG​GTC and LacI start-XbaI-RV CGTCT​AGAGAC​ACC​ATC​GAA​TGG​TGC​AAA​ACC). For the native shikimate kinase gene (Synpcc7942_0894, *aroK*) overexpression strategy, the gene was amplified from the purified *S. elongatus* PCC 7942 genome by touchdown PCR with the following primers: SK start-EcoRI-rv (CAT​GGAA​TTCGTG​TCA​TTG​GTG​ACA​GCT​CTG​AAC​GGC) and SK stop-NheI-fw (CAT​GGCT​AGCTTA​AGT​CTC​TAT​CTC​TGC​AGG​AGA​GCT​ATC​AGC). The amplified gene was cloned into pAM1579-LacI, containing the IPTG-inducible gene expression platform, kanamycin resistance and the homologous regions for the Neutral Site II insertion in *S. elongatus* genome (pAM1579-LacI-*aroK*, Figure 1B and Table 1). Any transformation was conducted by incubating *S. elongatus* with 0.5 µg DNA for 5 h in the darkness at 30°C. Then, bacteria were plated on the proper selection antibiotic (spectinomycin and/or kanamycin). To promote chromosomal segregation, the new colonies were periodically streaked on increasing concentration of selection antibiotic (until 100 μg/ml spectinomycin and/or 50 μg/ml kanamycin). The strains were confirmed by PCR and the full segregation was verified by using the following primers for Neutral Site I, NSI-fw AAG​CGC​TCC​GCA​TGG​ATC​TGA​C and NSI-rv GTA​GGG​ATT​TCG​CCA​GAT​CAA​TGC​C, and Neutral Site II, NSII-fw CGA​TCG​CTG​ACT​GAG​TTG​C and NSII-rv GAA​GCG​GGT​CAC​TAC​TTG​G.

### 2.3 qRT-PCR analysis

In order to validate the RNA-seq data, qRT-PCR analysis was performed. Briefly, pellets of 2PE_*aroK* mutant strain of *S. elongatus* PCC 7942 and the relative wild type strain grown under the different experimental conditions were obtained after centrifugation (4,000 *g*, 4°C, 20 min) of the respective liquid culture. Then, the pellets were freeze-dried and stored at −80°C until total RNA extraction was performed. Before performing nucleic acid extraction from the lyophilized pellets, they were washed twice with a 0.9% (w/v) NaCl solution and, in order to prevent bacterial RNA degrading, incubated with 1 ml of RNAprotect R Bacterial Reagent (Qiagen, Hilden, Germany) for 5 min and at room temperature. After centrifugation (5,000 *g*, 10 min, 4°C), total RNA extraction was performed by using RNeasy Mini Kit R (Qiagen, Hilden, Germany) as previously described (Campobenedetto et al., 2020). The amount of total RNA was measured with a UV/visible nano-spectrophotometer (BioSpec-nano, Shimadzu, Japan), and the quality was checked by 1% (w/v) agarose gel electrophoresis. The isolated RNA was then used as template for retro-transcription (cDNA Maxima H Minus First Strand, Thermo Fisher Scientific, United States), following the manufacturer’s instructions. The obtained cDNA was consequently used for quantitative real-time PCR using QuantStudio 3 (Thermo Fisher Scientific, United States), Maxima SYBR Green qPCR Master Mix (Thermo Fisher Scientific, United States), and the primers reported in Table 2. After evaluating the stability of each reference gene (*secA, ppc, prs,* and *16s*) under the different experimental conditions, the expression levels of the target genes (*kivD, adhA, aroG* ^*fbr*^ *, pheA* ^*fbr*^ *, aroK*) were calculated using the Pfaffl method (Mannino et al., 2021). Primers for both target and reference genes listed in Table 2 were designed with Primer3 software (Untergasser et al., 2012).

**TABLE 2:** Genes employed for qRT-PCR analysis. For each gene, the name, acronym, and nucleotide sequences for both Forward (F) and Reverse (R) primers are reported in the table.

Gene	Definition	Typology	Primer sequence (5'–3')		Size
			Forward	Reverse	
*secA*	preprotein translocase, SecA subunit	References Gene	ACG​ACG​GTC​AGA​TTG​CCG​AGA​T	GCG​ACA​TTC​CCT​GCT​GGA​TTA​G	204
*ppc*	phosphoenolpyruvate carboxylase	References Gene	CCC​TTG​CCA​GGA​CCA​GAT​GA	CGT​CGG​GTG​AGC​GGT​GAA​AA	146
*prs*	ribose-phosphate pyrophosphokinase	References Gene	TCT​TGC​CCT​ACT​ACG​GTT​ACG​C	TCG​CTC​CAG​CCT​GAG​TGA​TTA​G	247
*16s*	16s ribosomal RNA	References Gene	GCA​AGC​CTG​ACG​GAG​CAA​C	CGG​ACG​CTT​TAC​GCC​CAA​T	160
*kivD*	phenylpyruvate decarboxylase	Target Gene	TCA​TCA​TCA​ACA​ACG​ACG​GC	GCT​GGC​TAA​AGA​AGA​CGC​TC	240
*adhA*	alcohol dehydrogenase A	Target Gene	ACT​TTG​TTG​GGG​TGG​TGT​TG	ACT​ATG​GCT​GAG​CAC​TAC​CC	239
*aroG* ^*fbr*^	DAHP synthase, feedback-inhibition-resistant	Target Gene	CTG​ACG​TTT​GCC​AGC​AGA​TT	GCA​TTC​GCC​AGT​TGA​CGT​AA	184
*pheA* ^*fbr*^	chorismate mutase/prephenate dehydratase, feedback-inhibition-resistant	Target Gene	GCG​ACA​AAA​CTT​CAC​CCG​AT	CCA​GAC​GGG​GCA​TAA​TCA​GA	167
*aroK*	shikimate kinase	Target Gene	GCA​AAT​CGA​AAC​GCA​GGT​CT	CCG​GAG​CCT​CAG​TTT​GTA​GA	203

### 2.4 HPLC analysis

2-PE production by mutant strains was assessed by ultra-high-performance liquid chromatography (UHPLC UltiMate 3000, Thermo Fisher, Waltham, Massachusetts, United States) equipped with a Hypersil GOLD™ C18 reversed phase column (250 × 4.6 mm, 5 μm; Thermo Fisher Scientific) and UV detector set at 205 nm. As mobile phase, acetonitrile and H_2_O (50:50) was used with 1 ml/min flow rate, 40°C. L-phenylalanine was quantified through the Hypersil GOLD™ Amino column (120 × 4.6 mm, 5 μm; Thermo Fisher Scientific) at 210 nm. 9 mM sulfuric acid was pumped as mobile phase at 0.8 ml/min, column oven was set at 50°C. For sample preparation, 1 ml of bacterial culture was collected and centrifuged at 12,000 *g* for 10 min, and 20 µL of the supernatant was injected for HPLC analysis.

### 2.5 Whole-cell protein extraction

Ten mL cell cultures were collected before the gene expression induction and 5 days since IPTG supplementation. The cell culture was centrifuged at 4,000 g for 10 min and the cell pellet was twice washed with 0.9% (w/v) NaCl. Then, the cell pellet was depigmented from the chlorophyll a by washing it twice in 10 ml cold acetone and centrifuged at 4,000 *g* for 10 min. Next, the cell pellet was lyzed in 2 mM Tris-HCl, 0.4% (w/v) SDS, pH 7.6 and 100 mM DTT, incubated 30 min at 95°C and centrifuged at 15,000 g for 10 min. From the supernatant, whole-cell proteins were purified by following methanol-chloroform method (Wessel and Flügge, 1984). The protein pellet was resuspended in 10 mM Tris-HCl pH = 7 and 1% (w/v) SDS and the protein content was assessed by BCA method with Quantum Protein kit (Euroclone, Milan, Italy), following the manufacturer’s instructions. Proteins were resolved on 12% SDS-PAGE gel, by running at 80 V for 3 h.

### 2.6 Glycogen content determination

One mL of cell culture was collected before induction of the gene expression and at 1 days, 3 days and 6 days since supplementation of IPTG. The cell culture was centrifuged at 13,000 *g* for 10 min and processed as previously reported in (Osanai et al., 2011) with some modifications. Briefly, the cell pellet was lysed in 100 µL of 3.5% sulfuric acid and boiled for 40 min. Then, a centrifugation of 13,000 *g* for 2 min was followed by addition of 350 µL of 10% (w/v) trichloroacetic acid and incubated for 10 min at room temperature. The quantification was conducted from 150 µL of supernatant, by adding 750 µL of 6% (v/v) 2-aminotoluene (diluted in glacial acetic acid) and boiled for 10 min. The absorbance was measured at 635 nm. The standard curve was performed with D-glucose (0–3 mg/ml).

### 2.7 Bioinformatics search of class-specific enzymes

An alignment-based approach was employed to mine the *S. elongatus* PCC 7942 genome for genes encoding phenylalanine transaminases and enzymes able to catalyze the conversion of L-amino acids into α-keto-acids. The Enzyme Commission (EC) numbers EC 2.6.1.57, EC 1.4.99. B3, EC 1.4.1.5, and EC 1.4.3.2 were used to gather the amino acid sequences of, respectively, known aromatic amino acid transaminases, L-amino acid deaminases, L-amino acid dehydrogenases and L-amino acid oxidases from the UniProtKB database. Among the UniProtKB entries fulfilling the assignment to the EC numbers of interest, only reviewed records were selected with information extracted from literature and curator-evaluated computational analysis. Exception only for L-amino acid deaminases since no reviewed record was available in UniprotKB. For each functional category, an alignment of the protein sequences of known functional annotation was made against the protein sequences of *Synechococcus elongatus* PCC 7942 (taxid:1140) by using the blastp tool (default parameters). Alignment hits were ordered based on E-value and sequence percent identity. The unique hits in *Synechococcus elongatus* with at least an alignment with E-value < 0.001 were shortlisted. For each of the retained hits, the details of the top-scoring alignment were reported.

### 2.8 Metabolic effects of phenylalanine addition by metabolic model simulation

We used the GEnome-scale Metabolic model (GEM) iJB785 (Broddrick et al., 2016) publicly available for *Synechococcus elongatus* PCC 7942 in the BIGG database (King et al., 2016). The *S. elongatus* GEM iJB785 contains 768 metabolites, 849 reactions and 785 genes. The GEM was constrained with CO_2_ uptake rate and photon uptake rates derived from (Broddrick et al., 2019). The metabolic reconstruction was enriched with the reactions catalyzed by the heterologously expressed protein products of *kivD* and *adhA* genes as well as the exchange reaction corresponding to the 2-phenylethanol target product. To simulate the effects of medium supplementation with phenylalanine on the metabolic network, the corresponding exchange reaction was added to the metabolic reconstruction and Flux Variability Analysis (FVA) was carried out by maximizing biomass and comparing model predictions in the cases of zero or non-zero flux through the phenylalanine exchange reaction. By effect of FVA, each reaction was assigned a range of allowed fluxes in the two conditions. To gauge the effect of phenylalanine doping on the metabolic fluxes, for each reaction, the fraction (in percentage value) of allowed fluxes (not shared between the two conditions) was computed.

## 3 Results

### 3.1 Screening of several metabolite doping conditions to improve 2-phenylethanol production by p120 engineered *S. elongatus* PCC 7942

**FIGURE 2:**
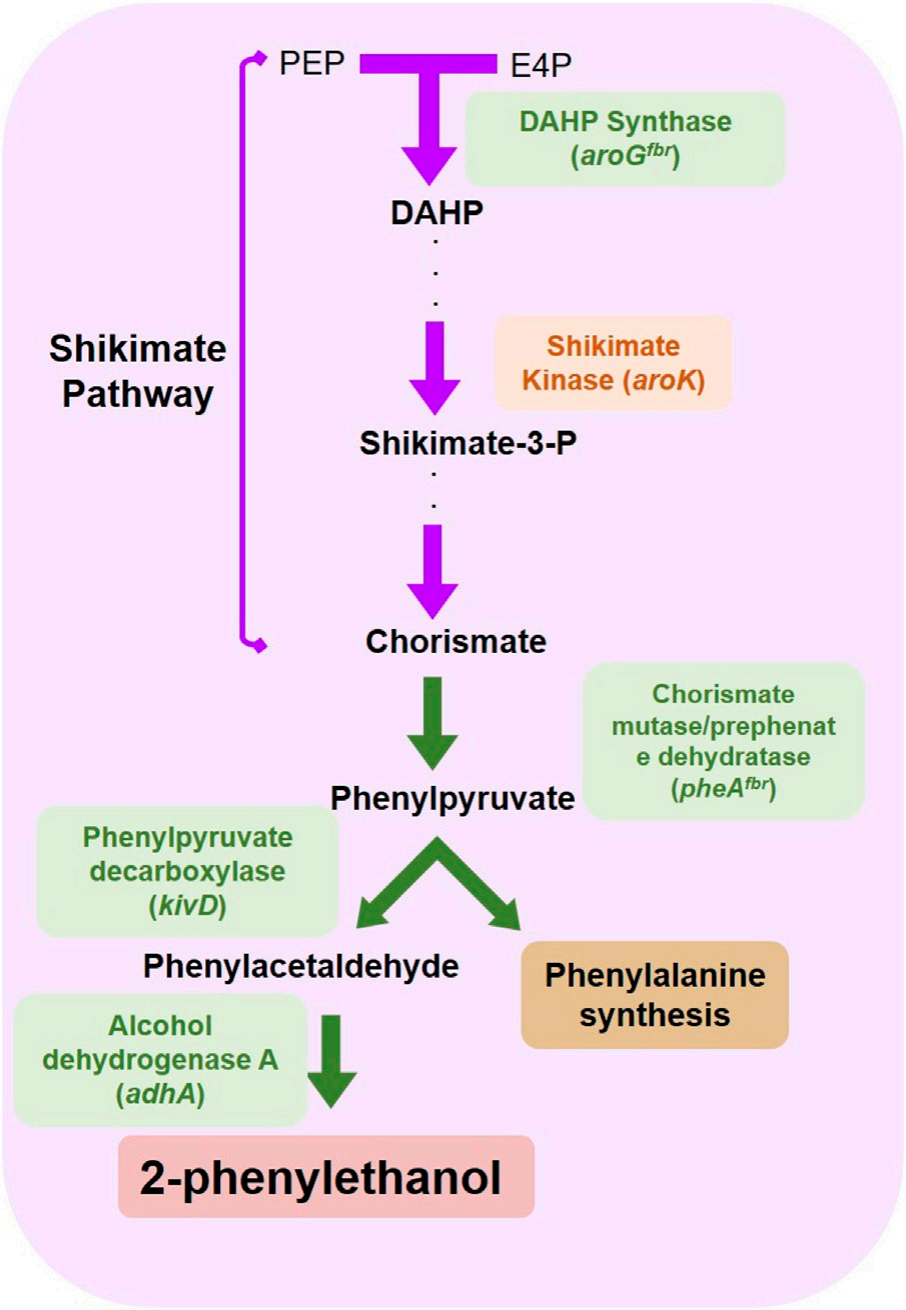
Overexpressed genes and relative protein products for 2-PE synthesis in *S. elongatus* PCC 7942 mutant strains p120 and 2PE_*aroK*. The reactions promoted by the p120 (green boxes) and 2PE_*aroK* (green and red boxes) mutant strains.

The non-native 2-PE synthesis pathway exploits some intermediates of the shikimate pathway, which is the biosynthetic route dedicated to the aromatics synthesis, such as aromatic amino acids and some vitamins (Braga and Faria, 2020). To understand whether the 2-PE synthetic pathway competes with other natural ones, we investigated the influence of some physiological aromatic molecules on the p120 *S. elongatus* PCC 7942 mutant strain metabolism. The p120 recombinant strain overexpressed four genes involved in 2-PE biosynthesis: *kivD*, *adhA*, *aroG* ^*fbr*^ and *pheA* ^*fbr*^ (Figure 1A). Their relative enzymatic role in 2-PE production was contextualized in Figure 2.

**FIGURE 3:**
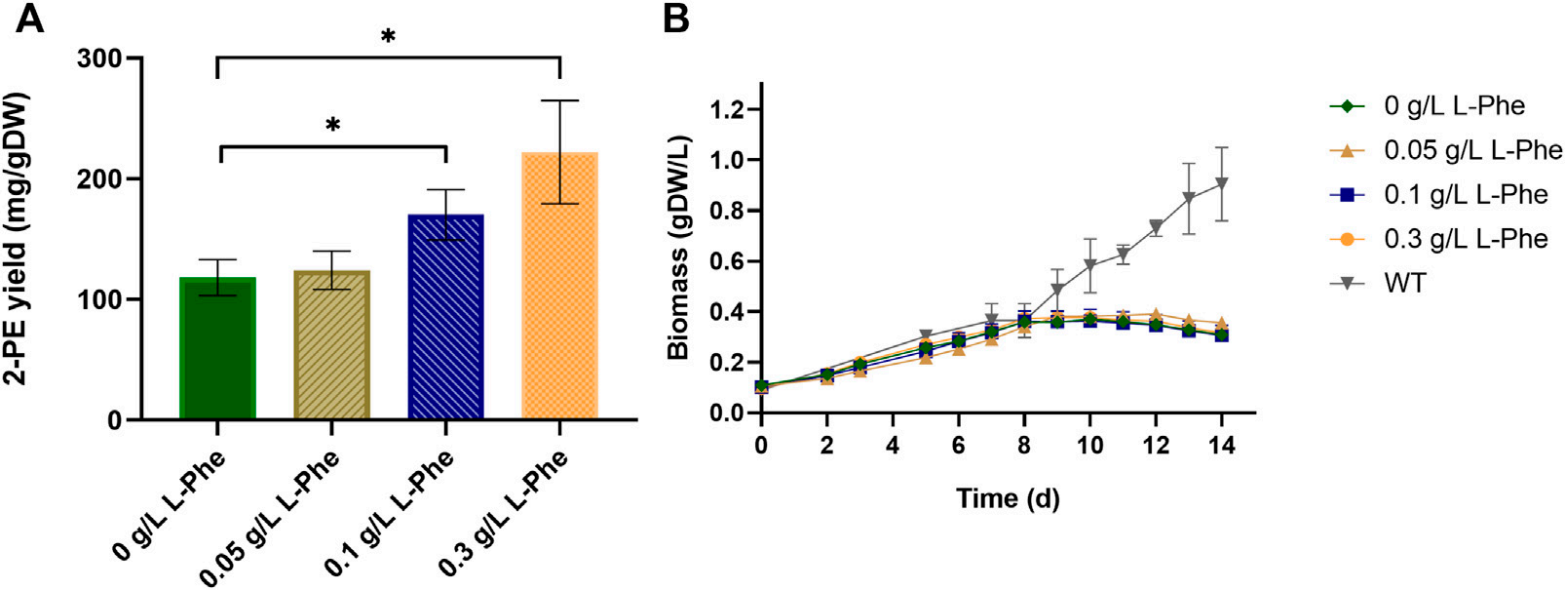
Effect of L-phenylalanine doping at three different concentrations on the p120 mutant strain. **(A)** The bar plot shows the 2-PE yield expressed as 2-PE mg produced normalized per g of dry cell weight (mg/gDW). The control condition is represented by the p120 mutant strain grown with no L-Phe. Asterisks indicate statistical significance for both 0.1 g/L and 0.3 g/L L-Phe doping versus control condition (unpaired two-tailed *t*-test, *p*-value < 0.05) and error bars report the standard deviation. **(B)** p120 mutant grown with L-Phe (0.05–0.3 g/L). As control conditions, p120 mutant (dark green) and wild type (grey) strains were both grown with no L-Phe. The L-Phe was added and gene expression was induced at the seventh day (about OD_730_ = 1, 0.26 gDW/L). The error bars show the standard deviation. Each test was conducted as three biological replicates.

First of all, the effect of IPTG addition was assessed for both wild type and p120 engineered strains. The wild type metabolism seemed not to be affected by the IPTG addition, indeed, no significant difference was noted from the relative growth curves (Supplementary Figure S1). By contrast, the addition of IPTG, which induces the gene expression of the heterologous pathway, to the p120 strongly influenced the mutant growth, causing a decrement in the growth rate with respect of the non-induced p120 strain (Supplementary Figure S1). Then, the three aromatic amino acids L-Phe, L-Tyr and L-Trp, and two aromatic-derived vitamins (folic acid and cyanocobalamin) were supplemented to the growth medium, either individually or in combination, at different concentrations. Each test was conducted in biological triplicate on the p120 mutant in shake flasks. Control condition represented the p120 recombinant strain grown with no L-Phe and all the results are shown in Figure 3 and Supplementary Figures S2,S3. The best performing conditions were the L-Phe supplementation out of the all tested compounds. Moreover, when assessing different L-Phe concentrations, we found that 0.3 g/L L-Phe almost doubled 2-PE yield (222 ± 42 2-PE mg normalized by g of dry cell weight capable of producing 2-PE, mg/gDW), in comparison with the yield of the reference condition (124 ± 15 mg/gDW, Figure 3A). However, the 2-PE final titer was not significantly increased (82 ± 11 mg/L at 12 days after the gene expression induction). Furthermore, L-Phe supplementation did not affect the bacterial growth, which appeared suffer the most from the gene expression induction, if compared with the growth kinetics of the wild type strain (Figure 3B). Further increasing L-Phe concentration in the medium beyond 0.3 g/L was ineffective, instead inhibited cell growth and led to chlorosis. The tested aromatic vitamins, i.e., folic acid and cyanocobalamin, did not affect neither bacterial growth nor 2-PE production, when supplemented into the medium singularly or in combination with L-Phe (Supplementary Figure S2). Also, L-Tyr and L-Trp showed no influence on the physiology of the p120 strain, when used individually as metabolite supplementation (data not shown). However, in combination with L-Phe, L-Tyr showed interference by negatively affecting 2-PE yield (Supplementary Figures S3B,F) and slightly increasing the growth rate (Supplementary Figure S3A). L-Trp only influenced the 2-PE production kinetics (Supplementary Figures S3G,H), by extending the production time, but had no significant effect on the 2-PE maximum yield (Supplementary Figure S3D). Due to these aromatics screening results, we chose to further explore the L-Phe supplementation as the only metabolite doping strategy in the present work. A deeper analysis about the fate of phenylalanine in *S. elongatus* mutant strains was performed and will be discussed in the next section.

### 3.2 Improving 2-phenylethanol production through metabolic engineering of the Shikimate Pathway

#### 3.2.1 Creation of 2-PE producing *S. elongatus* mutant overexpressing the endogenous shikimate kinase gene (2PE_*aroK*)

**FIGURE 4:**
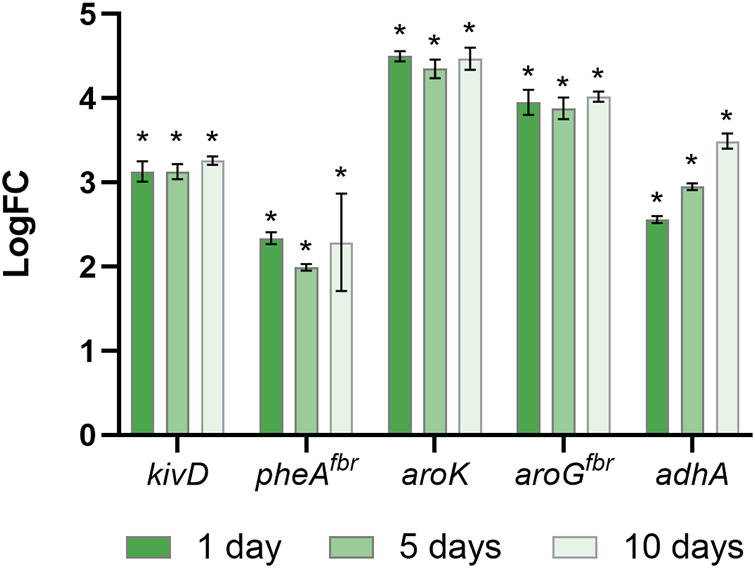
Temporal gene expression analysis. Gene expression of *kivD, adhA*, *pheA* ^*fbr*^ *, aroG* ^*fbr*^, *aroK* in 2PE_*aroK* cells after one, five and 10 days since gene expression induction. Data are expressed as log(2^^−∆∆Ct^) ± standard deviation. The symbol “*” indicates significant (*p* < 0.05) differences in gene expression of *kivD*, *adhA*, *pheA* ^*fbr*^, *aroG* ^*fbr*^, and *aroK* in 2PE_*aroK* cells after one, five or 10 days of induction respect to wild type condition, as calculated by student *t*-test.

To boost the carbon flow into SKP and to overcome some bottlenecks of this pathway, we co-expressed *aroG* ^*fbr*^, *pheA* ^*fbr*^ (from the p120 mutant strain) and the native Shikimate Kinase (as double gene copy of *SynPCC7942_0894*, aka *aroK*, into the Neutral Site II, under the control of IPTG-inducible promoter, Figure 1B), together with *kivD* and *adhA* to effectively produce 2-PE (Figure 2). Hence, the obtained mutant strain contains both p120 and *aroK* inserts, so, hereinafter, it is called 2PE_*aroK*. The successful obtainment of the mutant strain was first demonstrated by PCR screening (Supplementary Figures S4 A,C) and subsequently the accumulation of the protein corresponding to shikimate kinase following induction with IPTG was analyzed by SDS-PAGE analysis, where the band corresponding to the gene product of approximately 21 KDa was detected (Supplementary Figure S4B). Furthermore, an analysis about the expression related to all the five genes involved in 2-PE biosynthesis (*kivD*, *adhA*, *aroG*, *pheA* and *aroK*) was performed. The expression of all of the involved genes was confirmed by qRT-PCR at one, five and 10 days since the gene expression induction, keeping a stable expression level (Figure 4). Expression analysis on target genes was performed by first assessing the stability of reference genes under the different experimental conditions. Specifically, the expression of *secA, ppc, prs*, and *16s* genes was evaluated across both wild type strain and 2PE_*aroK* engineered strain after one, five and 10 days since gene expression induction (Supplementary Figure S5A). All the analyzed candidate reference genes were quite stable under the different experimental conditions. Among them, *secA* and *16s* genes had percentage coefficients of variation much less than 1%. Since *16s* gene encodes the most abundant transcripts in cyanobacteria, its expression had the lowest median Ct values (15.30 ± 0.08) compared with the other three candidates during qPCR amplification, despite the same amounts of total RNA and cDNA were used for qRT-PCR analysis On the other hand, the other candidates recorded very similar median Ct (*secA*: 19.00 ± 0.10; *ppc:* 19.56 ± 0.61; *prs:* 19.11 ± 0.52) (Supplementary Figures S5B). Consequently, also based on the percentage coefficient of variation, *secA* was chosen as the reference gene. This result is in agreement with the study of Xiao Luo et al. (2019) (Luo et al., 2019). By performing the statistical analysis with geNorm, NormFinder and BestKeeper softwares, *secA* and *prs* were found to be the most stable under all analyzed conditions, while *16s* could not be selected as a reference gene due to a significant imbalance in abundance between rRNA and mRNA molecules (Luo et al., 2019). Concerning target genes, the expression of 2PE_*aroK* engineered strain was assessed against the wild type, which, except for *aroK*, does not naturally express those heterologous genes (*kivD, adhA, pheA* ^*fbr*^, and *aroG* ^*fbr*^). Although a time-dependent change of target gene expression was not observed for most investigated genes, all the studied target genes were strongly upregulated in comparison with wild type condition (Figure 4). The double gene copy expression of *aroK* contributed to almost increase the *aroK* expression by 4.5-fold, over all the time tested, followed by *aroG* ^*fbr*^ (4-fold) and *kivD* (3.2-fold) (Figure 4). On the other hand, *pheA* ^*fbr*^ and *adhA* showed different profiles. Interestingly, *adhA* gene boosted its expression over the time, reaching a 3.5-fold increment at 10 days since the gene expression induction (Figure 4). *pheA* ^*fbr*^ resulted to be the less expressed gene, in all the tested conditions.

#### 3.2.2 Characterization of 2-PE producing *S. elongatus* mutant overexpressing the endogenous shikimate kinase gene (2PE_*aroK*)

**FIGURE 5:**
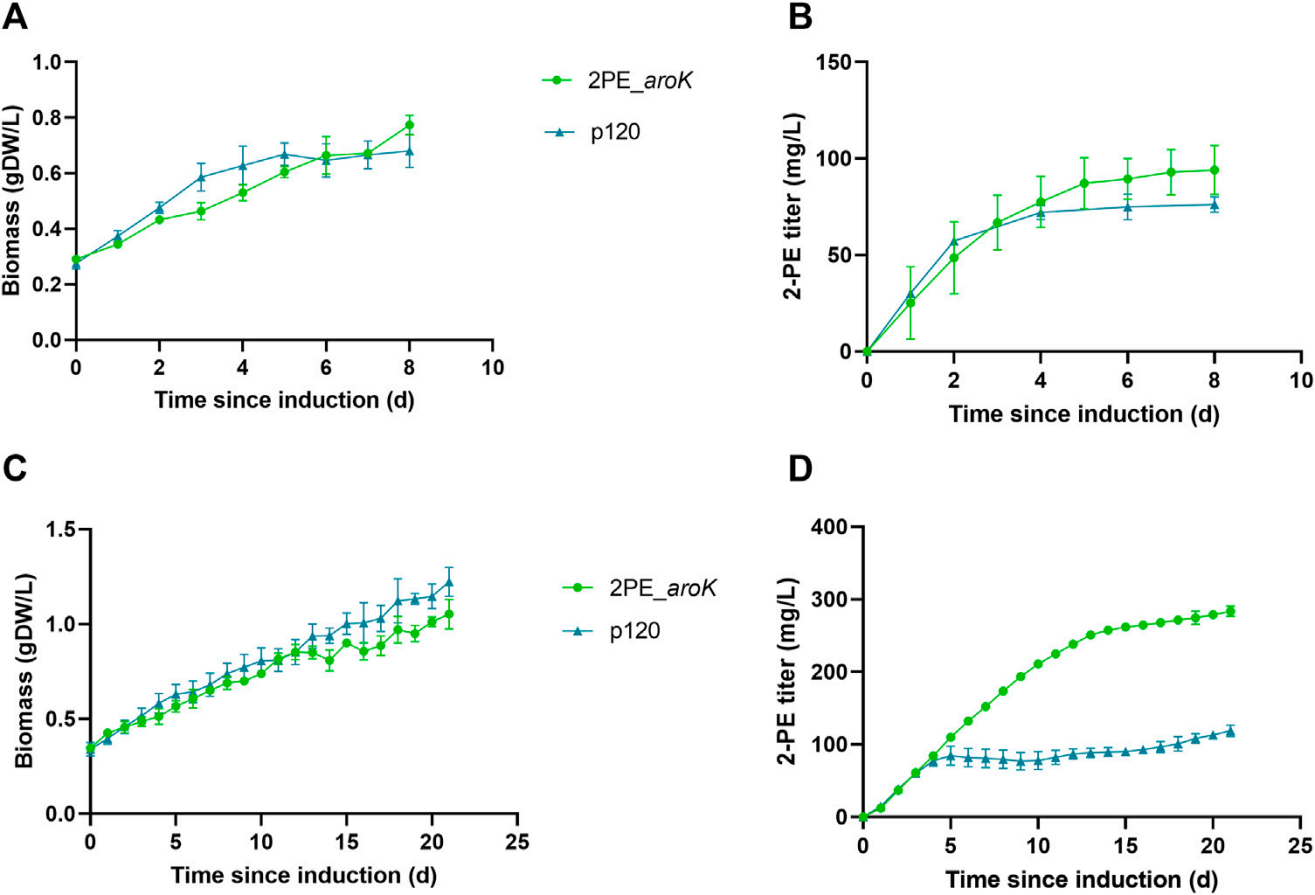
Characterization of 2PE_*aroK* engineered strain with or without L-Phe doping. Comparison between 2-PE production **(B,D)** and biomass formation **(A,C)** over the time since gene expression induction on 2PE_*aroK* under no L-Phe doping (top) or when exposed to L-Phe doping (bottom). As reference, the p120 strain was subjected to the same conditions. L-Phe was added and the gene expression was induced at around OD_730_ = 1 (0.26 gDW/L). Bars represent standard deviation. All tests were carried out in triplicate.

**FIGURE 6:**
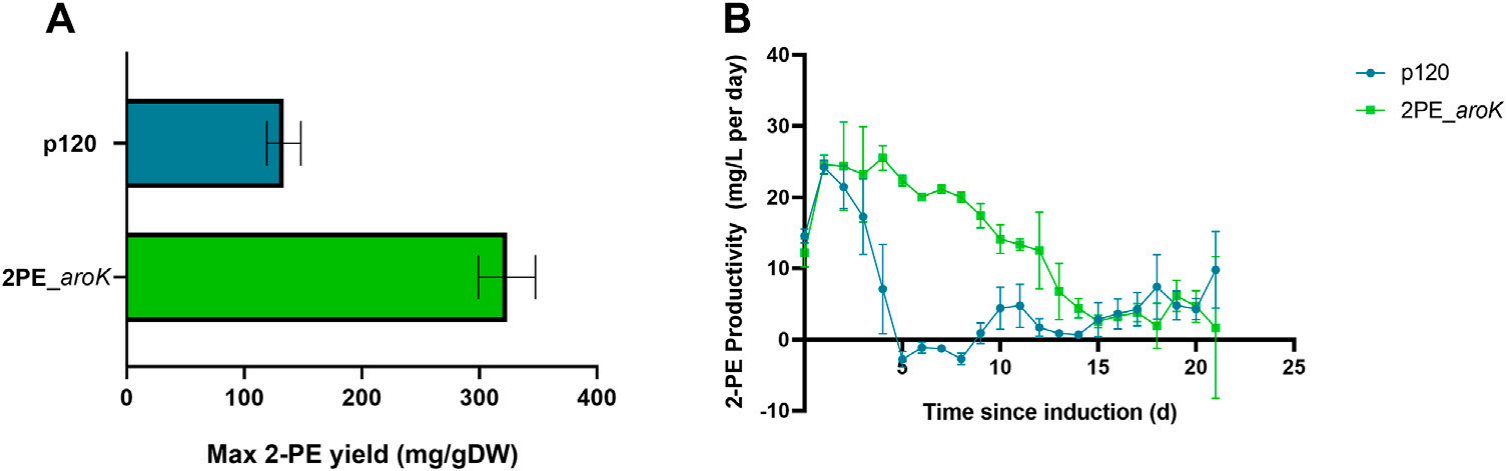
2-phenylethanol productivity in L-Phe-doped condition. **(A)** Maximum 2-PE mg normalized by the biomass produced during the experiment for both 2PE_*aroK* (green) and p120 (blue) strains. **(B)** 2-PE daily-produced, expressed as mg per liter per day, by 2PE_*aroK* (green) and p120 (blue) engineered strains over the time. The error bars represent standard deviation. All tests were conducted by means of three biological replicates.

2PE_*aroK* mutant strain was analyzed for its 2-PE production ability without L-Phe, as metabolite doping in growth medium. The abovementioned mutant strain reached a maximum production of 94.05 ± 12.6 mg/L after 8 days since gene expression induction (Figures 5A,B). Figures 5 C,D shows the characterization of 2PE_*aroK* mutant with supplementation of 0.3 g/L L-Phe, which has been previously shown to be the best metabolic doping condition, among those assayed. Thus, *aroK* overexpression combined with L-Phe doping strongly influenced the 2-PE production kinetics (Figure 5D). Specifically, the 2-PE production kinetics of 2PE_*aroK* strongly diverged from that of non-L-Phe-doped engineered strain and p120 mutant strain exposed to L-Phe doping, accordingly with our study (Figure 5), since four and 5 days after gene expression induction, respectively. Indeed, 2PE_*aroK* mutant reached a maximum titer of 285 ± 15 mg/L at the end of the experiment. However, 2PE_*aroK* reached the highest 2-PE yield at 15 days since induction, amounting to about 323 mg/gDW (Figure 6A) and corresponding to 263 ± 3 mg/L. Consequently, the daily 2-PE productivity was influenced (Figure 6B). Indeed, the 2PE_*aroK* produced on average 19.3 ± 4.86 mg/L per day, until 13 days since gene expression induction and then gradually decreased. Meanwhile, the reference p120 strain showed a significant 2-PE productivity until 5 days since induction (Figure 6B), reaching a maximum 2-PE yield of 133.65 ± 14.53 mg/gDW (Figure 6A). Also, the growth kinetics resulted to be affected, with a reduction in the specific growth rate (**µ**), equal to 0.09 days^−1^ and 0.12 days^−1^ for 2PE_*aroK* and p120 mutant strains, respectively. Table 3 precisely reports µ and the relative doubling time (D_t_) about 2PE_*aroK* and p120, with respect of our study. Additionally, the toxicity of 2-PE on *S. elongatus* PCC 7942 was assessed from 0.1 to 1 g/L, supplemented to the bacteria culture at OD_730_ = 1, mimicking the usual 2-PE production condition, to which the recombinant strain 2PE_*aroK* was subjected. However, no significant reduction in bacterial growth kinetics was observed at the 2-PE concentration obtained in the present investigation (about 0.3 g/L). However, higher 2-PE concentrations caused the bacterial growth reduced but not totally inhibited (Supplementary Figure S6).

**TABLE 3:** Specific Growth Rate (µ) and Doubling Time (Dt) of the p120 and 2PE_*aroK* strains. The µ was calculated in exponential growth phase, after gene expression induction and L-phenylalanine supplementation and it is expressed as day^−1^. SD, standard deviation.

Recombiant strain	Specific growth rate (d^−1^)	SD	Doubling time (d)	SD
p120	0.125	0.005	5.6	0.21
2PE_*aroK*	0.092	0.004	7.6	0.34

Furthermore, it is known that shikimate kinase uses ATP as co-substrate. To understand if the ATP pool necessary to sustain SK overexpression could come from the glycogen catabolism, beside photosynthesis, we quantified glycogen content from the biomass before and after one, three and 6 days gene expression induction. Glycogen quantification from 2PE_*aroK* biomass did not show any consumption after the gene expression induction, while the glycogen content steadily increased as the biomass grew (Supplementary Figure S7).

### 3.3 L-phenylalanine doping effect on mutant physiology

#### 3.3.1 L-phenylalanine consumption analysis

**FIGURE 7:**
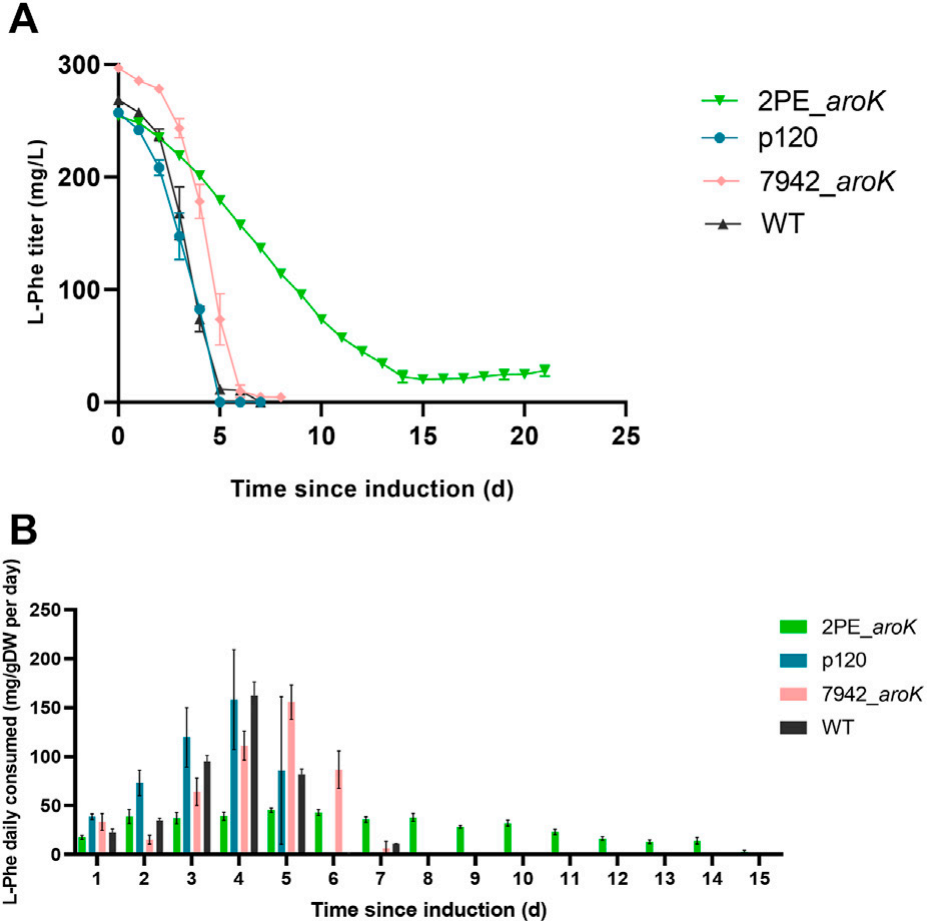
L-phenylalanine consumption analysis. **(A)** L-phenylalanine titer detected in the supernatant for 2PE_*aroK* (green), p120 (blue) and 7942_*aroK* (pink) mutant strains and the relative wild type *S. elongatus* PCC 7942 (grey). **(B)** The L-Phe daily consumed was normalized by the biomass produced. The results were expressed as mg of L-Phe consumed by g dry cell weight every day after the gene expression induction. The error bars represent standard deviation. All tests were carried out in triplicate.

The daily consumption of the supplemented L-phenylalanine in the growth medium was investigated for 2PE_*aroK* and p120 mutant strains, *S. elongatus* overexpressing only *aroK* gene (i.e., 7942_*aroK*) and the wild type *S. elongatus* as well (Figures 7A,B). The 2PE_*aroK* mutant gradually consumed L-Phe up to 14 days since induction, with a steady average consumption rate of 30 mg L-Phe/gDW per day (Figure 7B). In contrast, the p120 reference mutant and 7942_*aroK* consumed all available L-Phe within the first five and 6 days since induction, respectively. However, a basal L-Phe uptake by the wild type *S. elongatus* PCC 7942 exists. Although *S. elongatus* PCC 7942 can produce L-Phe by itself, it consumed all the available L-Phe in 7 days (Figures 7A,B) and drastically increased its consumption since 3 days after the L-Phe addition into the growth medium, which is a comparable trend detected with the p120 and 7942_*aroK* mutants.

#### 3.3.2 Bioinformatics analysis about L-phenylalanine consumption in 2-PE producing *S. elongatus* PCC 7942

**FIGURE 8:**
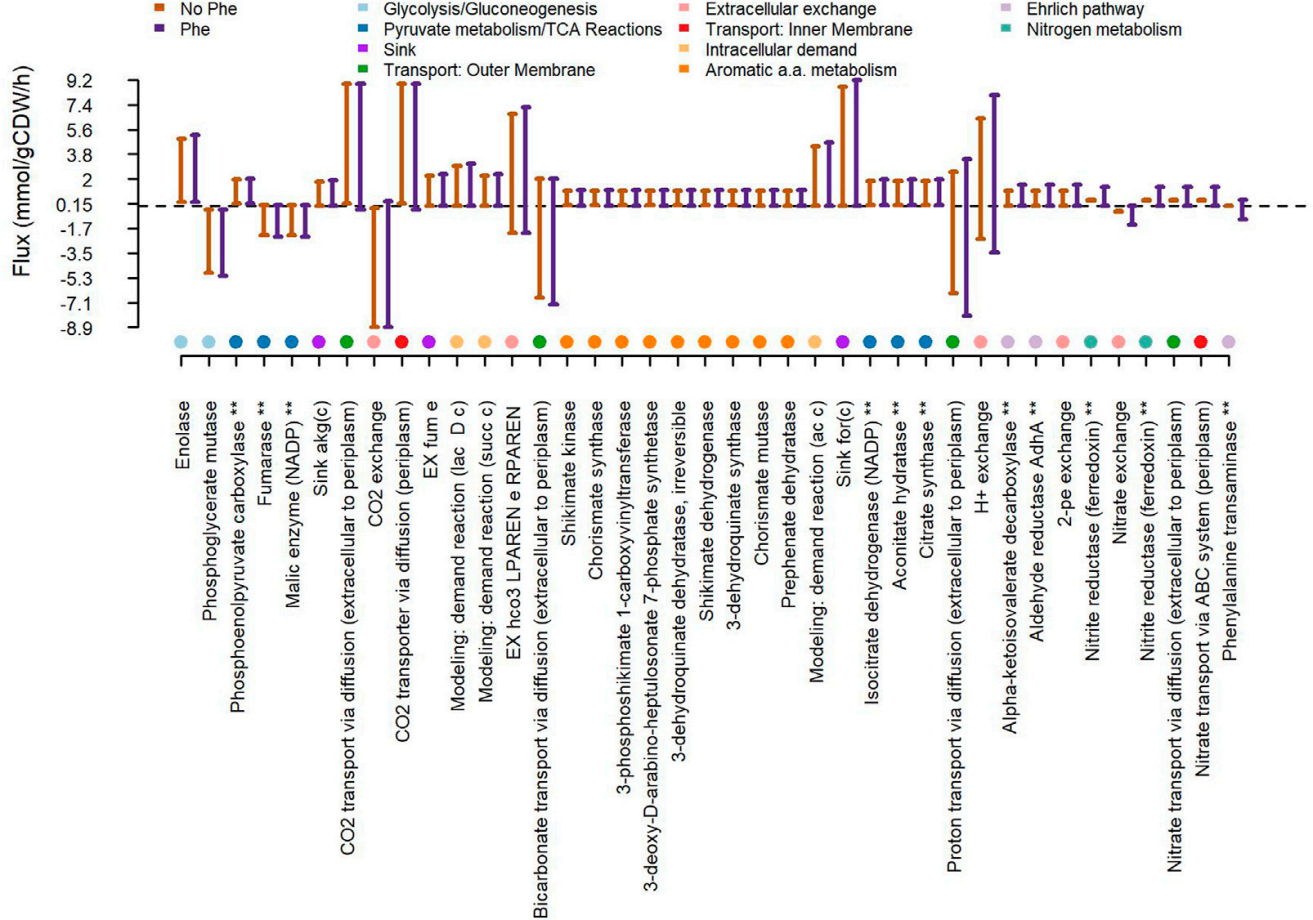
Effects of medium supplementation with L-phenylalanine on the metabolic network of the *S. elongatus* PCC 7942 mutant producing 2-phenylethanol. Flux variability analysis (FVA) was carried out in the absence/presence of Phe in the medium. Each reaction was assigned a range of allowed fluxes in the two conditions. For each reaction, the fraction (in percentage value) of allowed fluxes, which do not overlap between the two conditions, was computed. Shown are the individual reactions whose flux ranges in the presence/absence of phenylalanine differ by at least 5%. Reactions on the horizontal axis marked by “*” are mentioned in the main text.

The natural use of L-Phe for 2-PE biosynthesis has been widely investigated mainly in plants and yeast, while a few bacteria are known to naturally synthetize 2-PE (Zhang et al., 2014b; Liu et al., 2018a; Liu et al., 2018b). In yeast the 2-PE production is based on the Ehrlich pathway (Liu et al., 2019a; Gu et al., 2020; Dai et al., 2021). According to this pathway, L-Phe is transaminated to phenylpyruvate, where α-ketoglutarate is the acceptor of the amino group and converted into glutamate. Subsequently, phenylpyruvate is transformed into 2-PE, through sequential decarboxylation and reduction reactions (Hazelwood et al., 2008). Here, through bioinformatics analysis, we investigated the fate of the L-Phe into the mutant *S. elongatus* p120 and we verified the completeness of the enzyme set carrying out the Ehrlich pathway. Since, the mutant producing 2-PE heterologously overexpresses both the phenylpyruvate decarboxylase (*kivD* gene) and the alcohol dehydrogenase A (*adhA* gene), it could be capable of putatively processing the last two steps of the Ehrlich pathway. Thus, we searched in UniProtKB database the protein sequences of *S. elongatus* PCC 7942 for putative phenylalanine transaminases encoding genes, which could transaminate L-Phe in phenylpyruvate (Gu et al., 2020). We identified several hypothetical phenylalanine transaminase proteins based on amino acid sequence similarity with proteins known to carry out aromatic aminotransferase activity either in *Thermococcus litorali* (Andreotti et al., 1994) or in *Saccharomyces cerevisiae* (Iraqui et al., 1998), as reported in Supplementary Table S1. Furthermore, we carried out a genome-scale metabolic Flux Variability Analysis (FVA) on the GEnome-scale Metabolic model (GEM) iJB785 (Broddrick et al., 2016, 2019), customized to account for the heterologous expression of the *kivD* and *adhA* protein products in *S. elongatus*. We could confirm that the addition of phenylalanine led to increased flux through the reactions involved in the putative Ehrlich pathway, as shown in Supplementary Figure S8. Besides, additional reactions mainly related to nitrogen metabolism, aromatic amino acid biosynthesis and to the tricarboxylic acid (TCA) cycle were affected by phenylalanine doping (Figure 8, Supplementary Table S2). Alternatively, some bacteria, such as *Proteus* sp. can produce 2-PE by expressing an oxidative deaminase (Liu et al., 2018a), which converts L-Phe in phenylpyruvate. We then verified the presence of enzymes converting amino acids into α-keto-acids by sequence alignment against proteins assigned to relevant functional categories, namely L-amino acid deaminases, L-amino acid oxidases, and L-amino acid dehydrogenases (Nshimiyimana, Liu and Du, 2019). We could not detect any statistical significant hit of L-amino acid deaminase enzymes in *S. elongatus* PCC 7942, whereas we could detect several candidates for L-amino acid dehydrogenases and L-amino acid oxidases (Supplementary Table S3). In particular, the FAD-dependent oxidoreductase Synpcc7942_0369 was detected by sequence homology with the known L-amino acid dehydrogenase slr0782 in *Synechocystis* sp. PCC 6803 (Schriek et al., 2009). As to L-amino acid oxidases, our alignment-based search highlighted the FAD-dependent oxidoreductases Synpcc7942_0946 and Synpcc7942_0369, as previously recorded in the literature (Gau et al., 2007).

## 4 Discussion

Engineer cyanobacteria into cyano-factories for producing chemicals from solar energy, CO_2_ and water is an appealing chassis to address either environment issues or economical and sustainable bioproduction platforms. Furthermore, cyanobacteria are notorious for their genetic malleability and today many powerful genetic tools are available (Santos-Merino, Singh and Ducat, 2019; Sengupta et al., 2019; Wang, Gao and Yang, 2020; Zhou et al., 2022). Indeed, cyanobacteria have been widely metabolically engineered to produce a huge range of valuable molecules (Zhou and Li, 2010; Lai and Lan, 2015; Santos-Merino, Singh and Ducat, 2019). However, in engineered strains often the expression of heterologous pathways can compete with the native ones for precursors, intermediates, reducing agents and energy charge, by altering the natural metabolic flux. As a common consequence, the metabolic burden for the heterologous expression leads to imbalance the cellular activities due to the redirection of cellular resources to biomass formation and growth, as well as target products synthesis (Su et al., 2020; Kulagina et al., 2021; Kastberg et al., 2022). Here, to deeper investigate the impact of 2-phenylethanol heterologous pathway expression on *S. elongatus* p120 mutant strain (already published elsewhere (Ni et al., 2018)), we screened the effect of several native aromatic molecules (three AAAs, folic acid and cyanocobalamin) on mutant physiology related to aromatics biosynthesis (Figure 3 and Supplementary Figures S2,S3), by assessing bacterial growth and 2-PE production. Indeed, the 2-PE synthetic pathway steals precursors and intermediates from the shikimate pathway, the common route for aromatics biosynthesis, and AAAs synthesis. Unsurprisingly, the major influence was recorded for the L-phenylalanine supplementation, at 0.3 g/L (Figure 3). Thus, we almost doubled 2-PE yield, up to 222 mg/gDW, when we supplemented L-Phe to the induced cyanobacteria for the heterologous expression of the 2-PE synthetic pathway, but any particular increase in 2-PE concentration was recorded. The Phe addition is a consolidated strategy adopted to push 2-PE production by yeast, where the Ehrlich pathway is exploited (Liu et al., 2019b; Gu et al., 2020; Dai et al., 2021). The Ehrlich pathway is composed of three enzymatic steps, where L-Phe is firstly transaminated, in the presence of α-ketoglutarate, as amino group acceptor, to produce phenylpyruvate. Next, phenylpyruvate is decarboxylated in phenylacetaldehyde, then, is reduced in 2-PE, by a dehydrogenase (Hazelwood et al., 2008). In cyanobacteria, the ability of 2-PE biosynthesis has not been reported, as well as, the existence of the Ehrlich pathway. Nevertheless, the p120 mutant heterologously expresses a decarboxylase (*kivD* gene) and an alcohol dehydrogenase (*adhA* gene) (Figure 4), which could be responsible for the 2-PE production via non-shikimate-pathway-based route. It still has to be understood if *S. elongatus* PCC 7942 can express itself a specific enzyme capable of processing L-Phe in phenylpyruvate, i.e., the first reaction in the Ehrlich pathway. However, through bioinformatic analysis, we identified several putative phenylalanine transaminases, which requires α-ketoglutarate as co-substrate, in a 1:1 ratio with L-Phe. Interestingly, by genome-scale metabolic Flux Variability Analysis (FVA), we found several reactions affected by L-Phe addition (Figure 8, Supplementary Table S2), including the Tricarboxylic acid Cycle (TCA). Among the several steps of this pathway, the reaction mediated by the isocitrate dehydrogenase, which catalyzes the production of α-ketoglutarate, is influenced by L-Phe doping. Accordingly, it could be possible that this increased flow through the TCA cycle effectively feeds the 2-PE production from L-Phe. Additionally, we pointed out an alternative pathway, previously highlighted in 2-PE producing bacteria (Hazelwood et al., 2008), which is equally noteworthy. In particular, by *in silico* homology search of protein sequences, we could gather preliminary evidence of hypothetical oxidative deaminases that can convert L-Phe in phenylpyruvate. Here, we identified in the FAD-dependent oxidoreductases Synpcc7942_0946 and Synpcc7942_0369 (Gau et al., 2007) two possible protein candidates to carry out L-amino acid oxidase activity. Noteworthy, the wild type strain of *S. elongatus* PCC 7942 showed itself a basal L-Phe uptake (Figure 7). In some cyanobacteria, a Phenylalanine ammonia lyase (PAL) activity has been detected (Moffitt et al., 2007; Babaoğlu Aydaş, Ozturk and Aslım, 2013). PAL catalyzes the non-oxidative deamination of phenylalanine to trans-cinnamate, the main branch for phenylpropanoid metabolism. Although, in *S. elongatus* PCC 7942 a PAL activity has not been reported so far, it could be presumable a similar trend, where the supplemented L-Phe could be directed towards this secondary metabolism. However, our findings suggest an important, although not pivotal, role of Phe in *S. elongatus* PCC 7942 physiology and could help to formulate hypothesis about the fate into the aromatics metabolism in cyanobacteria, but further studies are needed.

Additionally, we looked at some possible carbon flux limiting steps for the shikimate pathway. Besides cyanobacteria, several metabolic engineering attempts have already been conducted to enhance the carbon flow into the SKP. Commonly, the main strategies include: 1) the engineering of transport systems (mainly for carbohydrate-using microorganisms) (Báez-Viveros et al., 2004; Ahn et al., 2011; Gottlieb, Albermann and Sprenger, 2014; Gu et al., 2017), 2) the deletion of competing pathways (Báez-Viveros et al., 2004; Xu et al., 2017) or 3) optimization of the critical enzymes activity (Báez-Viveros et al., 2004). One of the most consolidated approaches concerns the very first step of the SKP, i.e., the condensation of intermediates from glycolysis and pentosephosphate-pathway, phosphoenolpyruvate (PEP), and erythrose-4-phosphate (E4P), respectively. This reaction is catalyzed by 3-deoxy-D-arabino-heptulosonate 7-phosphate (DAHP) synthase. *E. coli* encodes three DAHP synthase isoenzymes, which are inhibited by phenylalanine, tyrosine or tryptophan, respectively. Several mutational analyses highlighted different genetic approaches to overcome this feedback inhibition (Kikuchi, Tsujimoto and Kurahashi, 1997; Jiang et al., 2000; Jossek, Bongaerts and Sprenger, 2001; Liao et al., 2001; Hu et al., 2003; Chávez-Béjar et al., 2012) in order to increase the precursors input towards the SKP. Along with this, in *E. coli* another carbon flow-controlling step is related to the enzyme chorismate mutase, which catalyzes the release of chorismate from the SKP, the main branch point for the synthesis of aromatics. Also, the chorismate mutase is affected by a feedback inhibition by Phe or Tyr and, accordingly, *tyrA* ^*fbr*^ and *pheA* ^*fbr*^ mutated proteins have been created (Turnbull and Morrison, 1990; Vincent et al., 2002; Lütke-Eversloh and Stephanopoulos, 2005; Zhou et al., 2010; Cao et al., 2016). Nowadays, the co-overexpression of both feedback-inhibition resistant *E. coli* enzymes is common to gain aromatics production, mostly in *E. coli* and yeast (Báez-Viveros et al., 2004; Zhang et al., 2014a; Rodriguez et al., 2014, 2015; Wang et al., 2019; Koma et al., 2020), as well as in cyanobacteria (Ni et al., 2018; Brey et al., 2020; Deshpande, Vue and Morgan, 2020). Additionally, in the literature another reaction has been reported as high flux controlling step (Luttik et al., 2008; Takai A, Nishi R, Joe Y, 2009; Darmawi et al., 2012; Rodriguez et al., 2015; Xu et al., 2019), i.e. shikimate kinase-mediated reaction, the conversion of shikimate and ATP in shikimate-3-phosphate and ADP. For all the above reasons, we co-overexpressed *aroG* ^*fbr*^, *pheA* ^*fbr*^, from *E. coli*, and the native shikimate kinase (*SynPCC7942_0894*, *aroK*), to overcome the abovementioned bottlenecks of this pathway, together with *kivD* and *adhA* to synthesize 2-PE through *S. elongatus* PCC 7942 (Figure 2). The resulted 2PE_*aroK* mutant was able to express all the genes involved in 2-PE biosynthesis, keeping their expression stable (Figure 4). In particular, *aroK* resulted to be the most expressed gene, among the other ones, up to 4.5-fold higher than the wild type strain. The resulting mutant strain effectively increased 2-PE production in L-Phe doping condition (Figure 5). It is interesting to remind that SK overexpression in the absence of L-Phe supplementation was not significantly effective on the 2-PE production kinetics (Figure 5). Under phenylalanine doping condition, with respect of our study (Figure 5) on p120 engineered strain (Ni et al., 2018), the 2PE_*aroK* mutant rose (by 2.2-fold) 2-PE production up to 285 mg/L, keeping a steady daily productivity of 19 mg/L for the first 2 weeks (Figure 6B). Interestingly, the 2-PE productivity was higher within 9 days since gene expression induction, although the genes expression resulted quite stable over the time (Figure 4). Probably, different mechanisms rules the 2-PE production, maybe at the proteins level or turn over events.

Moreover, the 2PE_*aroK* decreased its growth kinetics, by 1.4-fold. This detrimental effect on the growth kinetics could be more likely due to the metabolic focus on the target compound overproduction. Indeed, the toxicity assay carried out on *S. elongatus* PCC 7942 exposed to several 2-PE concentrations (0.1–1 g/L), confirmed that no negative effects are caused by 2-PE at the concentration produced by 2PE_*aroK* recombinant strain, equal to about 0.3 g/L (Supplementary Figure S6). An inherent link between SK overexpression and L-Phe supplementation was further supported by the different L-Phe consumption profile between p120, 7942_*aroK* and the wild type strains when compared to 2PE_*aroK* (Figure 7). Indeed, the 2-PE producing mutant overexpressing *aroK* gene reduced the bacterial L-Phe consumption rate, which remained steady for almost 2 weeks, and simultaneously improved the 2-PE production, compared to the p120 engineered strain.

Interestingly, shikimate kinase uses ATP as co-substrate and its functional overexpression in a biological system is not obvious, due to the fine energy and ATP homeostasis through the metabolic network. Besides the photosynthesis, another ATP source in cyanobacteria comes from the catabolism of glycogen, the major carbon storage compound in many cyanobacteria species. Nevertheless, our glycogen quantification from 2PE_*aroK* biomass did not show any consumption after the gene expression induction, where the glycogen content steadily increased as the biomass grew (Supplementary Figure S7), up to 12% of cell biomass, as previously reported (Hasunuma et al., 2013; De Porcellinis, Frigaard and Sakuragi, 2017).

Additionally, the maximum theoretical yield for the 2-PE (C8) producible from Phe (C9) is roughly 0.9 mol/mol. Here, for 2PE_*aroK* mutant, the maximum yield would be higher than the theoretical one (≈150%), which further suggests that the 2-PE cannot be produced only from the Phe, but from photosynthesis as well. Moreover, the SK enzyme is placed upstream the putative pathway processing Phe into 2-PE, therefore it is reasonable to speculate that a combinatorial and synergistic effect of multiple phenomena on the 2PE_*aroK* mutant aromatics metabolism could exist, whose relative interconnections are still unclear but worthy of further investigation. Nevertheless, our approach demonstrates how the complex microbial response to metabolic perturbations, could be fixed by setting up *ad-hoc* cultivation processes for any specific metabolic engineering applications.

## 5 Conclusion

In this study, a recombinant photosynthetic cell factory producing 2-phenylethanol was successfully improved. Overall, this investigation highlighted the potentiality of a combinatorial strategy, which can be able to overcome synergistically different metabolism-related issues, by combing metabolic engineering and process engineering. Hence, we firstly explored the metabolite doping strategy, by adopting a phenylalanine-supplemented BG11 medium. Here, we supplied the main C source (CO_2_) and L-phenylalanine at different times during the bacterial phototrophic cultivation. As a result, we led to segregate growth and 2-PE production phases and increase the final 2-PE concentration and yield. Secondly, the implementation of the native shikimate kinase overexpressed, in addition to the 2-PE synthetic pathway, modified the cell kinetics by both increasing the 2-PE amount and reducing the phenylalanine consumption rate during the mutant strain growth. Finally, the L-Phe doping growth condition with this new mutant strain led to a maximum 2-PE titer of 285 mg/L, about 2.4-fold higher than previously reported in the literature (Ni et al., 2018) and, to the best of our knowledge, the highest 2-PE production reported so far in photoautotrophic growth condition for photosynthetic microorganisms. Here, the overexpression of the native shikimate kinase firmly suggests a bottleneck in the SKP of cyanobacteria, indicating this enzyme as suitable target gene for many others aromatics and derived compounds production strategies.

From an application-oriented point of view, the impressive increment in 2-PE production and yield achieved in the present work could support the implementation of L-Phe-doped BG11 medium, also for a large-scale production. Although, single substrate feeding is often conventionally favored in biotechnological applications, it could lead to multiple metabolic constraints, mainly due to the fine coordination required to simultaneously balance global cell metabolism, growth and overproduction of target compounds (Babel, 2009; Liu, Santala and Stephanopoulos, 2020; Hartline et al., 2021). On the other hand, several experimental efforts have already demonstrated that is possible overcome these limitations by adopting metabolite doping or co-substrate feeding, accordingly with particular metabolic engineering applications (Papazi et al., 2012; Anfelt et al., 2015; Liu et al., 2019a; Park et al., 2019; Gu et al., 2020; Ndayisenga et al., 2021), in agreement with the present work. Additionally, thanks to the very poor nutritional requirements of cyanobacteria, the BG11 growth medium is still suitable for further supplementation, in the light of large-scale application. Furthermore, the adoption of phenylalanine-rich waste waters, such as dairy wastes, has the potential to further reduce the process costs, making this 2-PE production cyano-based system sustainable and feasible.

In conclusion, although this photosynthetic cyanobacterial factory is still at lab-scale, our study provides the basis to further optimize this biotechnological process for 2-PE production. Hence, by finely regulating the CO_2_ and L-Phe supply and light intensity, it could be possible to simultaneously increase both the growth and biosynthetic kinetics and productivity for 2-PE. Furthermore, by exploring how this mutant strain processes phenylalanine could help not only the optimization of 2-PE biosynthesis, but also the whole aromatics production, and stimulate in-depth studies about the still poorly characterized aromatic-related pathways in cyanobacteria and their interconnection.

## Data Availability

The raw data supporting the conclusions of this article will be made available by the authors, without undue reservation.

## Abbreviations

2-PE, 2-phenylethanol; AAA, aromatic amino acid; *aroK*, gene encoding for Shikimate Kinase; DAHP, 3-deoxy-D-arabino-heptulosonate 7-phosphate; fbr, feedback-inhibition resistant; IPTG, Isopropyl β-D-1-thiogalactopyranoside; Phe, phenylalanine; SK, shikimate kinase; SKP, shikimate pathway.
