# Supplementary material for: Combining metabolite doping and metabolic engineering to improve 2-phenylethanol production by engineered cyanobacteria
Source: Front Bioeng Biotechnol. 2022 Sep 20;10:1005960. doi: 10.3389/fbioe.2022.1005960 (PMC9530348; doi:10.3389/fbioe.2022.1005960)
Supplement: Supplementary file 4 [file DataSheet1.docx]

**Combining metabolite doping and metabolic engineering to improve 2-phenylethanol production by engineered cyanobacteria**

**Giulia Usai^1,2^, Alessandro Cordara^1*^, Angela Re^1,x^, Maria Francesca Polli^1,3^, Giuseppe Mannino^4^, Cinzia Margherita Bertea^4^, Debora Fino^2^, Candido Fabrizio Pirri^1,2^, Barbara Menin^1,y^**

^1^Centre for Sustainable Future Technologies, Fondazione Istituto Italiano di Tecnologia, Via Livorno 60, 10144, Turin, Italy.

^2^ Department of Applied Science and Technology - DISAT, Politecnico di Torino, Corso Duca degli Abruzzi 24, 10129, Turin, Italy.

^3^ Department of Agricultural, Forest and Food Sciences - DISAFA, University of Turin, 10095, Grugliasco, Italy.

^4^ Plant Physiology Unit. Department of Life Sciences and Systems Biology, University of Turin. Via Quarello 15/A, 10135 Torino, Italy

^x^ Present address: Department of Applied Science and Technology - DISAT, Politecnico di Torino, Corso Duca degli Abruzzi 24, 10129, Turin, Italy.

^y^ Present address: Istituto di Biologia e Biotecnologia Agraria, Consiglio Nazionale delle Ricerche IBBA-CNR, Via Alfonso Corti 12, 20133 Milano, Italy.

***Correspondence**:

Alessandro Cordara

[alessandro.cordara@iit.it](mailto:alessandro.cordara@iit.it)

***Supplementary Material***

***
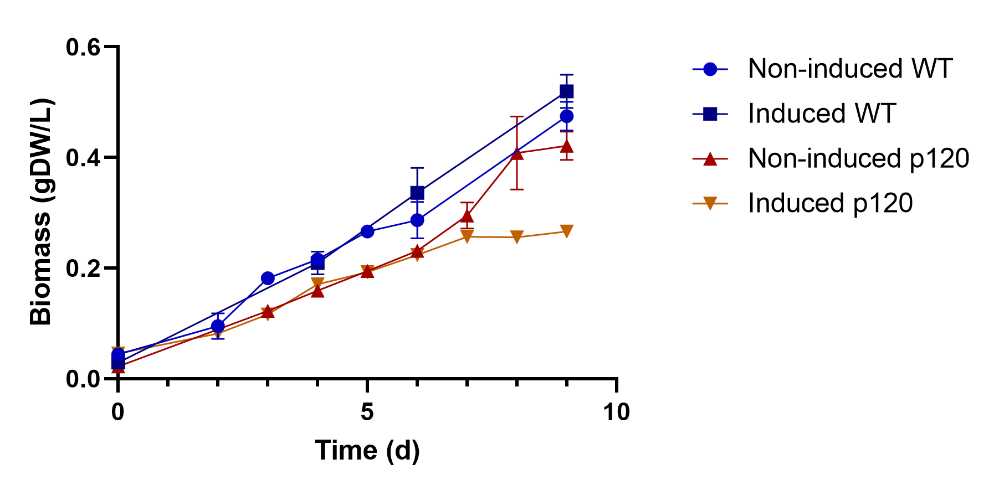
***

**Supplementary Figure 1. Effect of IPTG addition for gene expression induction.** The growth kinetics, expressed as g of dry cell weight per liter, of both wild-type *S. elongatus* and the recombinant p120 strain subjected or not to IPTG. All tests were conducted by means of three biological replicates.

*
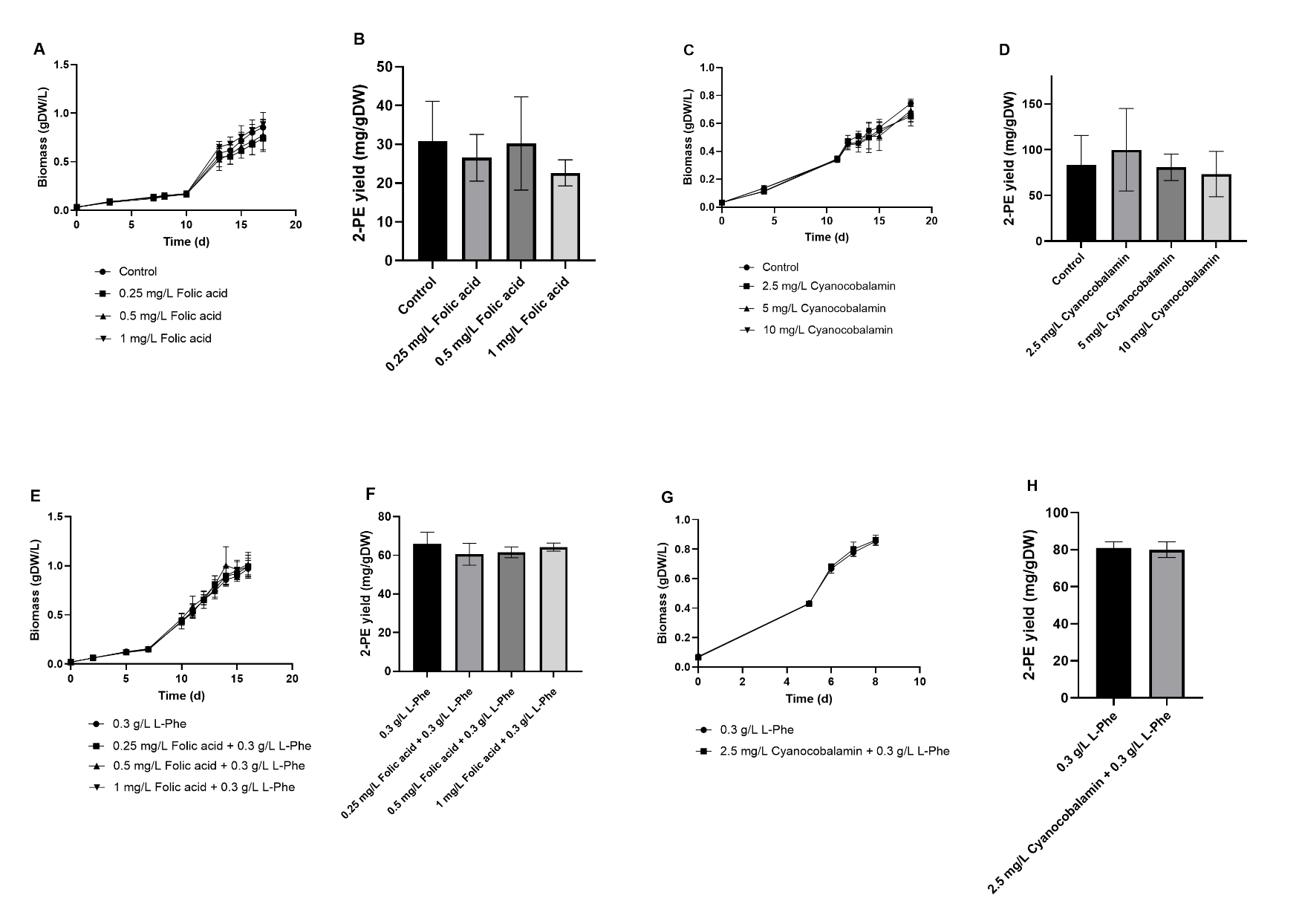
*

**Supplementary Figure 2. Response of *S. elongatus* p120 strain growth and 2-PE production to the supplementation in BG11 medium of two aromatic vitamins**. All the results are means of three biological replicates and are relative of a reference condition (Control or 0.3 g/L L-Phe). **A**, **C** and **B**, **D** represent the p120 growth curve (gDW/L) and 2-PE yield (mg/gDW), respectively, at three different concentrations of folic acid or cyanocobalamin. **E**, **G** and **F**, **H** represent the p120 growth curve and 2-PE yield, respectively, when folic acid and cyanocobalamin were supplemented in combination with 0.3 g/L L-Phe. Statistical analyses were conducted as one-way ANOVA or unpaired two-tailed t-test (p-value < 0.05).


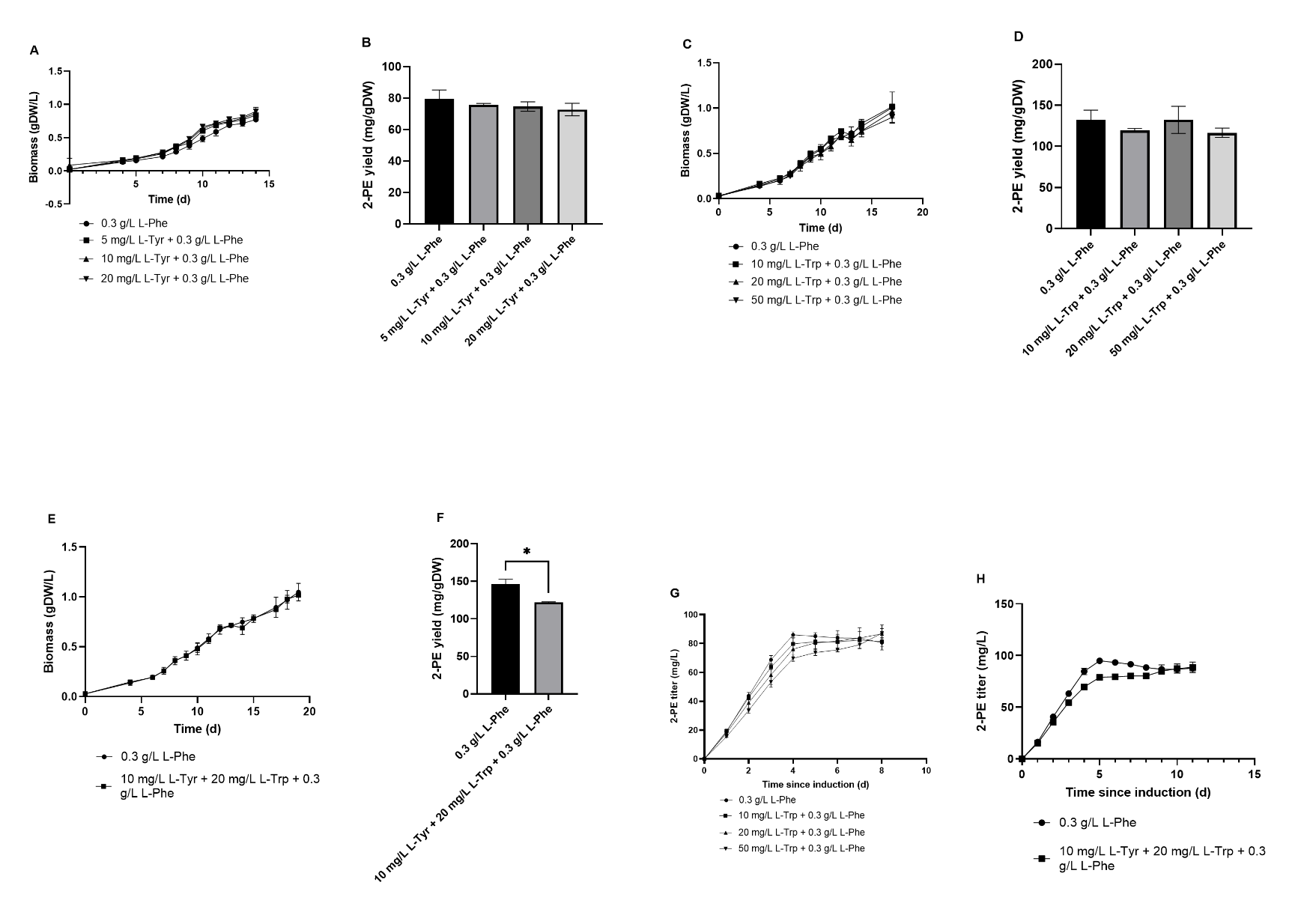


**Supplementary Figure 3. Response of *S. elongatus* p120 strain growth and 2-PE production to the supplementation in BG11 medium of aromatic amino acids**. All the results are means of three biological replicates and are relative of a reference condition (0.3 g/L L-Phe). **A**, **C** and **B**, **D** represent the p120 growth curve (gDW/L) and 2-PE yield (mg/gDW), respectively, at three different concentrations of L-Tyr or L-Trp, when supplemented together with 0.3 g/L L-Phe. **E**, **F** represent the p120 growth curve and 2-PE yield, respectively, in a combination of the three aromatic amino acids condition. **G**, **H** show the 2-PE production kinetics in the presence of L-Phe and L-Trp or all the three aromatic amino acids. Statistical analyses were conducted as one-way ANOVA or Unpaired Two-tailed t-test (p-value < 0.05). Asterisk shows a statistical significance.


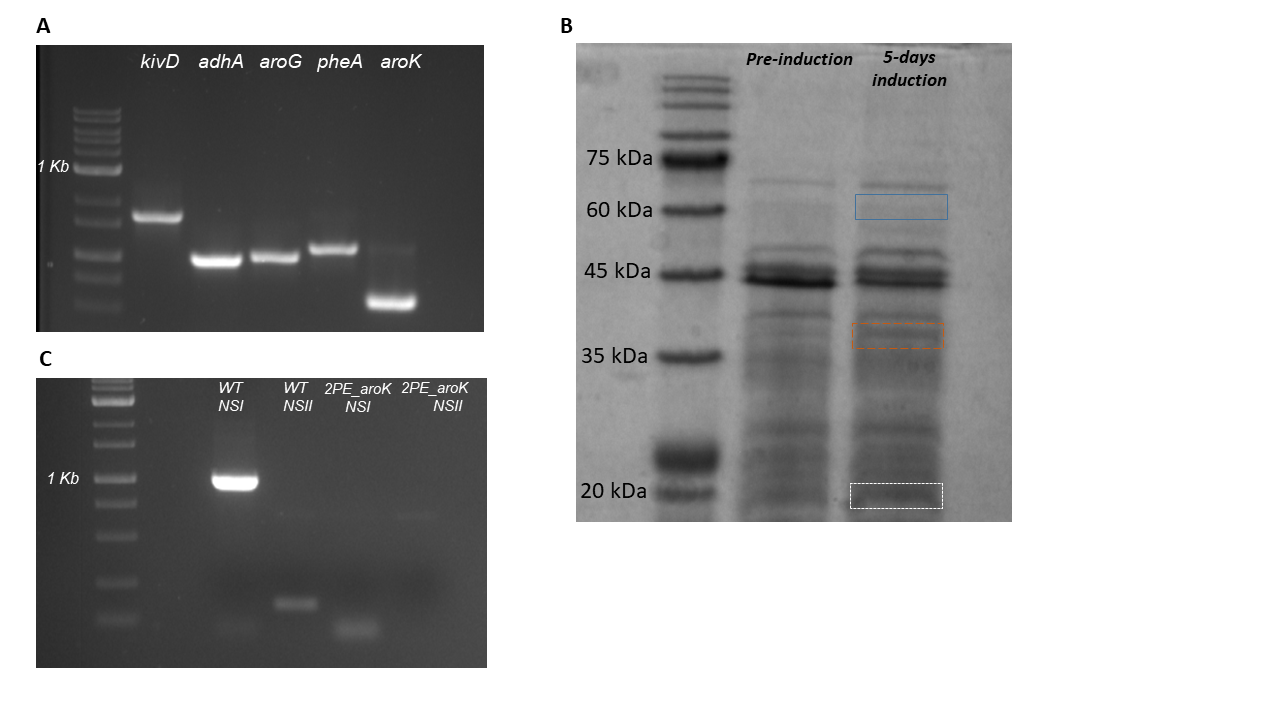


**Supplementary Figure 4**. **Genetic and Protein profile of 2PE_*aroK* mutant**. **A)** Gene profile of 2PE_*aroK* mutant. *kivD*, 1647 bp; *adhA*, 1011 bp; *aroG^fbr^*, 1065 bp; *pheA^fbr^*, 1161 bp; *aroK*, 573 bp. **B**) Whole-cell protein profile of 2PE_*aroK* strain. 30 µg of proteins were loaded for both pre-induction and 5 days after induction samples. Phenylpyruvate decarboxylase, ≈ 61 kDa, blue box; 3-deoxy-D-arabino-heptulosonate 7-phosphate (DAHP) synthase, 38.5 kDa, and alcohol dehydrogenase A, ≈ 36 kDa, could be both part of the dashed box. Shikimate kinase, ≈ 21 kDa, white dotted box. The bifunctional chorismate mutase/prephenate dehydrogenase is ≈ 43 kDa could be included into the most intense band zone, due to endogenous proteins, which make it difficult to be distinguished. All five genes are under the control of IPTG-inducible promoter, which suffers from leaky expression, so it is possible to have a slight expression of the target proteins even before the actual gene expression induction. C) Segregation evaluation. The wild type NSI (1005 bp) and NSII (168 bp) were amplified in PCR. In 2PE_*aroK* strain the same sites were not amplified, because occupied by p120 insert and the *aroK* gene, respectively, indicating a complete segregation of the inserts in each *S. elongatus* chromosome.


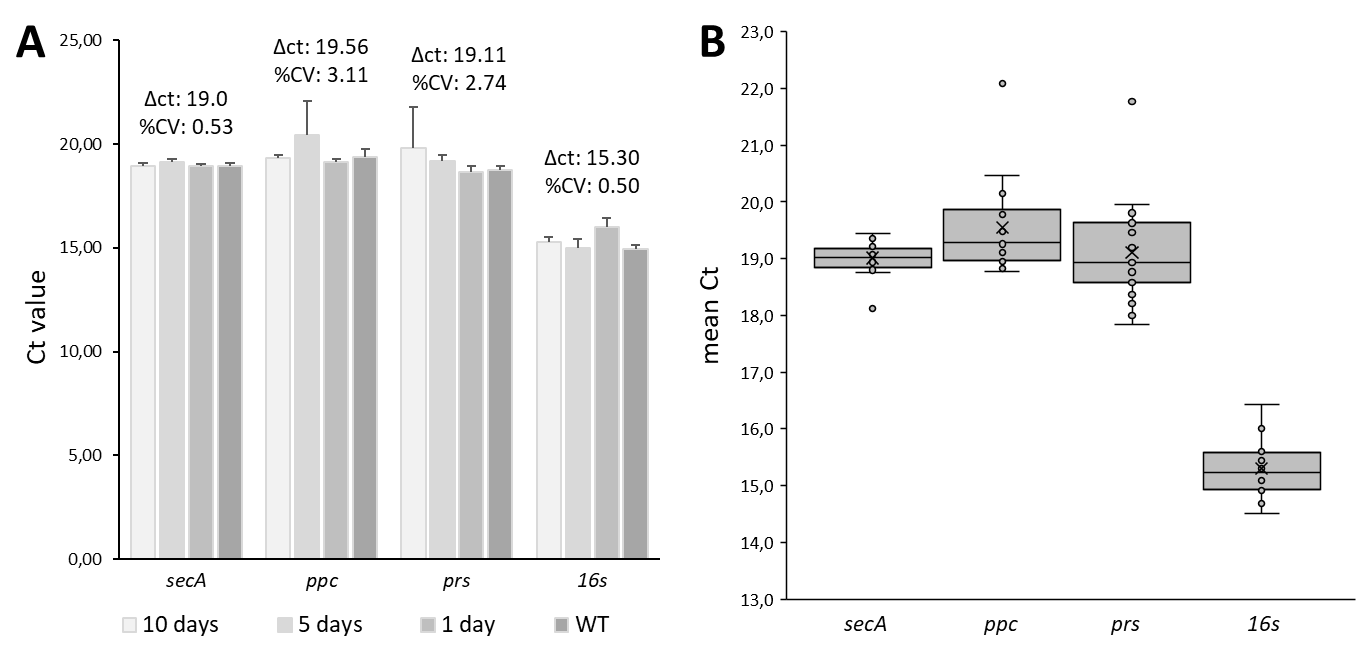


**Supplementary Figure 5.** **qRT-PCR analysis of reference genes of *S. elongatus* PCC 7942**. A) range of cycle threshold (Ct) values of the four reference genes used in the present work (*secA, ppc, prs,* and *16s*) across wild-type and 2PE_*aroK* engineered strain after one, five and ten days since gene expression induction, and the relative wild-type strain of *S. elongatus* PCC 7942. B) range of cycle threshold (Ct) values of the four candidate reference genes across all samples of *S. elongatus* PCC 7942. The box indicates the Ct value within the 25th and 75th percentiles. Whiskers include the Ct values in the 10th and 90th percentiles. The line across the box represents the median.


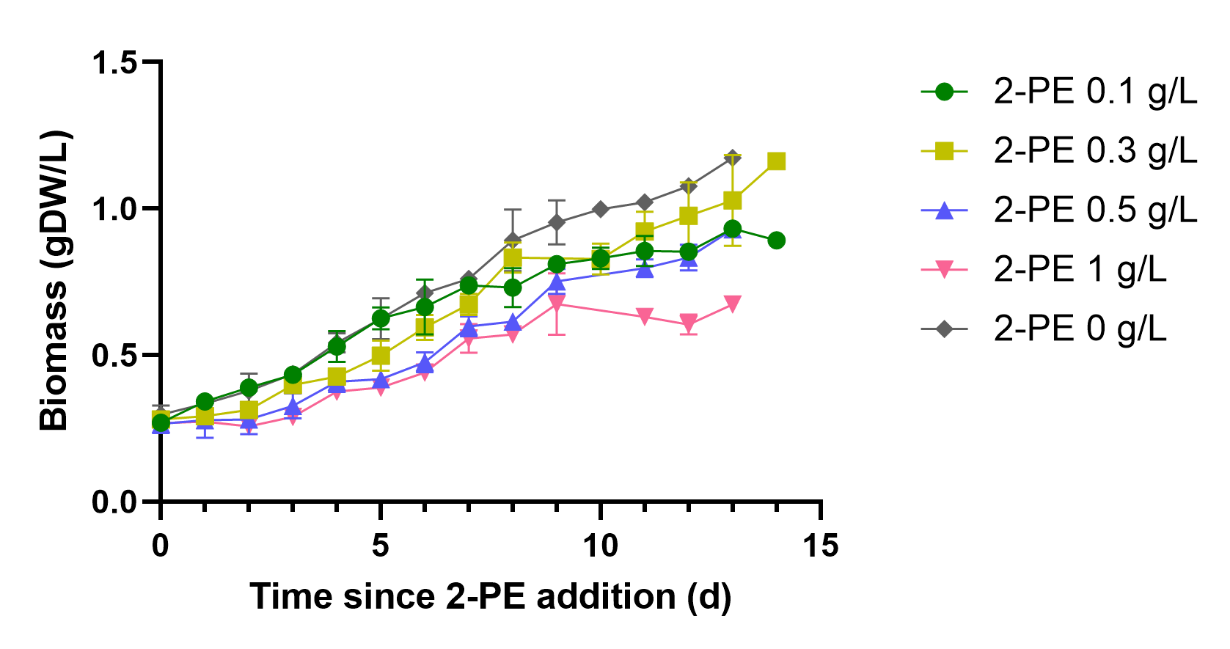


**Supplementary Figure 6. Evaluation of toxicic effect of 2-phenylethanol on *S. elongatus PCC 7942***. Four increasing 2-PE concentration were tested, from 0.1 to 1 g/L. 2-phenylethanol was added to the medium at around OD_730_ = 1 (0.26 gDW/L). Bars represent standard deviation. All tests were carried out in biological triplicate.


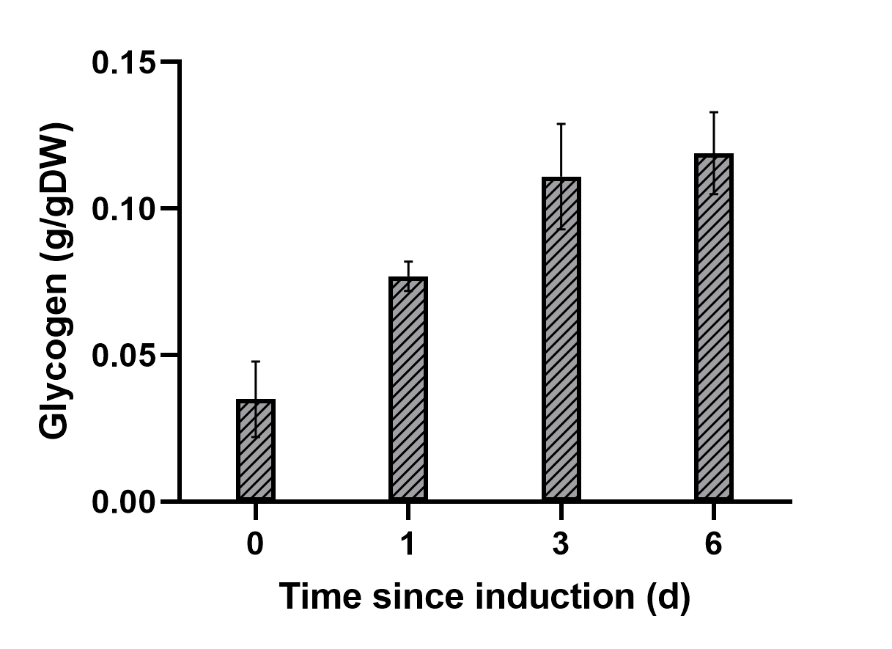


**Supplementary Figure 7. Glycogen content in 2PE_*aroK* mutant before and after the gene expression induction**. The glycogen content is reported as g of glycogen per g of cell dry weight (g/gDW). Bars represent standard deviation. All tests were carried on in biological triplicate.


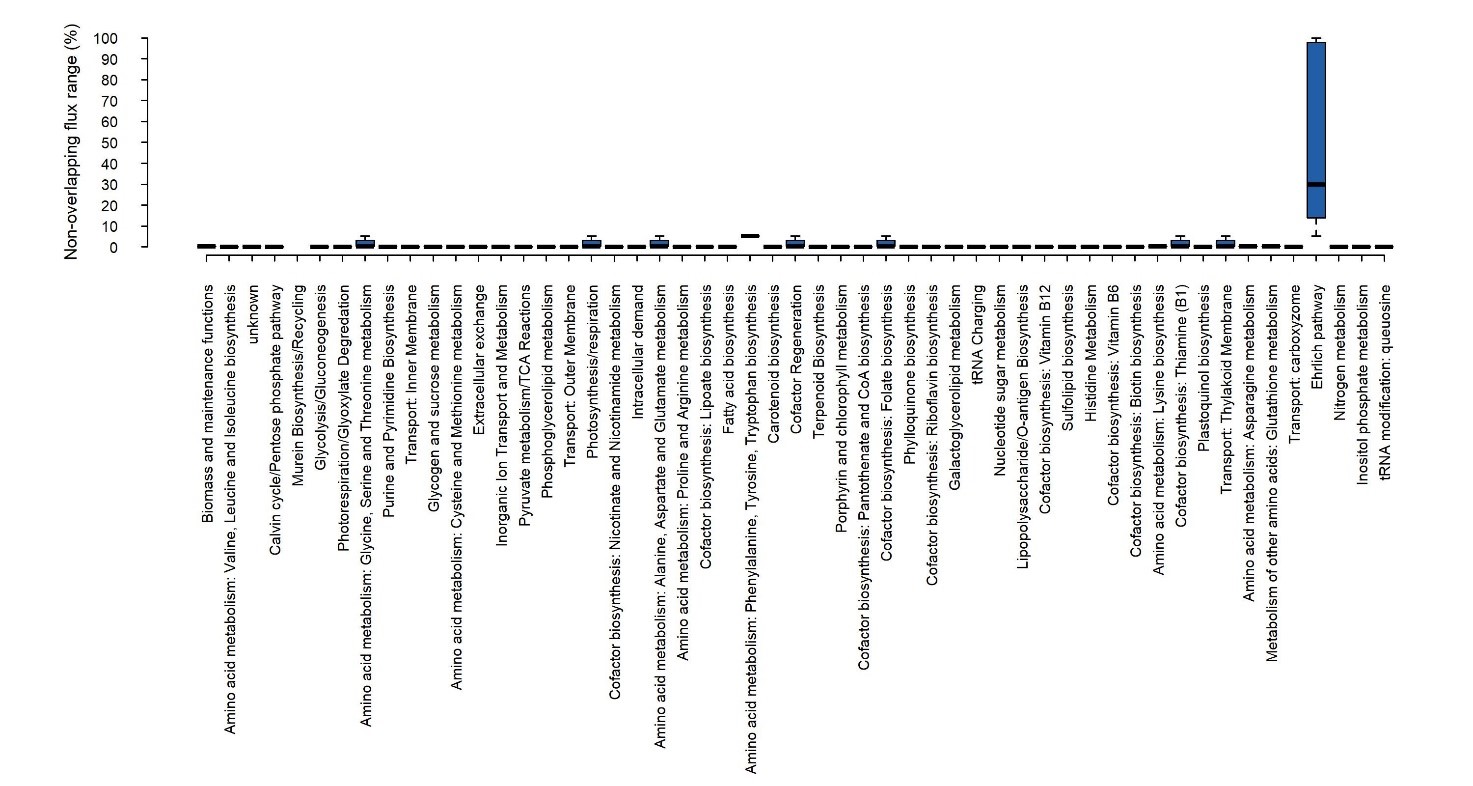


**Supplementary Figure 8.** **Reactions of the metabolic reconstruction of the 2-PE-producing strain affected by medium supplementation with phenylalanine.** L-phenylalanine supplementation affects mainly the fluxes through the reactions of the Ehrlich pathway. Shown is the simulated effect of phenylalanine addition to the medium on different functional categories. Flux variability analysis (FVA) was carried out in the absence/presence of Phe in the medium. Each reaction is assigned a range of allowed fluxes in the two conditions. For each reaction, the fraction (in percentage value) of allowed fluxes, which do not overlap between the two conditions, is computed. Shown is the distribution of the non-overlapping flux ranges of the reactions catalysed by the enzymes belonging to different functional categories.

**Supplementary Table 1 (.xls).**  **Alignment-based analysis of the *S. elongatus* PCC 7942 genome for genes encoding phenylalanine transaminases**. Alignment hits with statistical significance (E-value < 0.001) resulting from the alignment of known aromatic amino acid transaminases against the amino acid sequences of *Synechococcus elongatus* PCC 7942**.** For each retained hit, the table displays the scoring details corresponding to its top-scoring alignment. The table provides functional annotations retrieved from the UniProtKB database, both for the query and the hit proteins.

**Supplementary Table 2 (.xls)**. **Effects of** **medium supplementation with phenylalanine on the metabolic reactions of the *S. elongatus* PCC 7942 mutant producing 2-phenylethanol**. Flux variability analysis (FVA) was carried out in the absence/presence of phenylalanine in the medium. Each reaction is assigned a range of allowed fluxes in the two conditions. For each reaction, the fraction (in percentage value) of allowed fluxes, which do not overlap between the two conditions, is computed. Shown are the individual reactions whose flux ranges in the presence/absence of phenylalanine differ by at least 5%.

**Supplementary Table 3 (.xls). Alignment-based analysis of the *S. elongatus* PCC 7942 genome for genes encoding L-amino acid deaminases, L-amino acid dehydrogenases and L-amino acid oxidases**. Alignment hits with statistical significance (E-value < 0.001) resulting from the alignment of known L-amino acid deaminases, dehydrogenases and oxidases against the amino acid sequences of *S. elongatus* PCC 7942. For each retained hit, the table displays the scoring details corresponding to its top-scoring alignment. The table provides functional annotations retrieved from the UniProtKB database, both for the query and the hit proteins. (*) The query gene is a known L-amino acid dehydrogenase in *Synechocystics* (Schriek *et al.*, 2009). (**) Two genes known as L-amino acid oxidase in *Synechococcus elongatus* (Gau *et al.*, 2007).
